# Characterization of serum and tissue oxytocinase and tissue oxytocin in the pregnant and non-pregnant mare

**DOI:** 10.1038/s41598-023-31540-9

**Published:** 2023-03-21

**Authors:** Mariana Diel de Amorim, Lynn Dong, Michael Byron, Robert A. Foster, Claudia Klein, Monique Saleh, Tarek Saleh, Claire Card

**Affiliations:** 1 Department of Clinical Sciences, Cornell University, 930 Campus Rd, Ithaca, NY 14853 USA; 2 Immunopathology Research and Development Laboratory, Department of Biomedical Sciences, Cornell University, 930 Campus Rd, Ithaca, NY 14853 USA; 3 Department of Pathobiology, Ontario Veterinary College, University of Guelph, 50 Stone Road E., Guelph, ON N1G 2W1 Canada; 4 Department of Veterinary Clinical and Diagnostic Science, University of Calgary, 2500 University Drive NW, Calgary, AB T2N 1N4 Canada; 5 Department of Biomedical Sciences, Ontario Veterinary College, University of Guelph, 50 Stone Road E., Guelph, ON N1G 2W1 Canada; 6 Department of Large Animal Clinical Sciences, Western College of Veterinary Medicine, 52 Campus Drive, Saskatoon, SK S7N 5B4 Canada; 7 Present Address: Translational Pathology, Bristol Myers Squibb, Route 206 and Provinceline Rd., Princeton, NJ 08543 USA; 8 Present Address: Federal Research Institute for Animal Health, Institute of Farm Animal Genetics, Hoeltystr.10, 31535 Neustadt-Mariense, Germany

**Keywords:** Gonads, Pituitary gland, Reproductive biology

## Abstract

Oxytocin is a hormone with functions in: reproduction, maternal bonding, milk ejection, and feeding/social behavior, and is reported to be present in a variety of tissues. Our goal is to characterize oxytocin and leucyl and cystinyl aminopeptidase (LNPEP/oxytocinase), a key regulator of oxytocin in mares. We measured serum and tissue LNPEP by ELISA from ovulation (D0) until D21–22 in non-pregnant (n = 5) and pregnant mares (n = 6); and in periparturient and postpartum mares (n = 18). Placenta (n = 7) and homogenized tissue of diestrus mares (n = 6) were evaluated using protein determinations and LNPEP ELISAs. Identification of LNPEP and OXT protein in tissues was also performed via western blot, immunohistochemistry and liquid chromatography-mass spectrometry (LC-MS/MS). Furthermore, in situ hybridization was performed for LNPEP and OXT on endometrium, myometrium, pituitary and corpus luteum (CL). Serum LNPEP concentration were similar. Placental LNPEP U/mg protein was highest in the body and pregnant horn. The highest to lowest LNPEP U/mg protein by tissue were: myometrium > follicle wall > endometrium > kidney > CL > liver. Oxytocin was identified in the equine pituitary, CL and placenta and is likely to act in autocrine or paracrine manner, while LNPEP may act systemically and locally to regulate the availability of OXT.

## Introduction

Oxytocin is a nonapeptide that is considered a neurohypophysial hormone. The majority of the oxytocin is synthetized in the magnocellular neurosecretory cells in the hypothalamic supraoptic and paraventricular nuclei; however, it is produced as a prepro-hormone. The prohormone is cleaved by neuroendocrine convertase to form oxytocin/neurophysin—1 which is packed into neurosecretory vesicles, transported and stored in Herring bodies at the axon terminals in the distal part of the neurohypophysis. Oxytocin/neurophysin—1 is released by exocytosis, and oxytocin is dissociated from neurophysin or is cleaved from neurophysin after release from the neurohypophysis^1^. Oxytocin in some species is also synthetized in other tissues such as the endometrium, myometrium, placenta, and corpus luteum^2^. It has many physiological roles, such as: myometrial contractility, parturition, luteolysis, maternal bonding, milk ejection and also modulation of social behavior^1,2^. Oxytocin administration elicits prostaglandin release after Day 10 in non-pregnant but not pregnant mares^3^. The equine endometrium expresses oxytocin/neurophysin—1 as identified by mRNA analysis^4^ and using immunoelectron microscopy^5^; however, the equine CL was not reported to be a source of oxytocin^6^. Additionally, the serial administration of oxytocin to mares in the mid luteal phase causes prolonged luteal function but this was affected by the timing, duration, dose, formulation and route of administration^7–9^. Further knowledge of oxytocin metabolism and its roles in the equine luteolysis and pregnancy is warranted.

Oxytocin is metabolized by aminopeptidase and carboxyaminopeptidase in serum and in various tissues by oxytocinase^10^. A variety of terms are applied to oxytocinase, including: placental leucine aminopeptidase (P-LAP), Cystine/Cystinyl aminopeptidase (CAP), and leucyl and cystinyl aminopeptidase (LNPEP)^11^. In rats, the protein called insulin-regulated aminopeptidase (IRAP) is the homologue to the human oxytocinase^12^. Oxytocinases are important in the body for homeostasis and are involved in the regulation of important physiologic processes such as blood pressure, as it regulates oxytocin, vasopressin and angiotensin^10,13^. Oxytocinase inactivates the oxytocin hormone by breaking the peptide bond between the N-terminal cysteine and adjacent tyrosine; and it is co-localized with the insulin responsive glucose transporter 4 (GLUT4) in the GLUT4 specialized intracellular vesicles^14^. The coupling of oxytocin (OXT) with its receptor (OXTR) increases LNPEP activity^15^. In vascular endothelial cells oxytocin stimulates the translocation of LNPEP via a protein kinase C dependent pathway coupled to the OXTR^15^.

Oxytocinase is ubiquitous to a wide array to tissues, including: the heart, skeletal muscle, adipocytes, vascular endothelial cells, small intestine, kidney, brain, testis, lung, liver and placenta^11,16–18^. In the placenta, LNPEP is present in the chorionic microvilli^19,20^, and the concentration of LNPEP was almost undetectable in the serum of non-pregnant women, but increased in the maternal blood during pregnancy, reaching a tenfold rise by the third trimester of pregnancy^18^. Oxytocinase is suggested to be responsible for maintaining homeostasis in pregnancy^18,21^, as pregnant women with pre-eclampsia or pre-term delivery have abnormal levels of LNPEP^22,23^. The metalloprotease ADAM12 was reported to recognize the amino acid sequence for the conversion of the membrane form of LNPEP to the soluble form^24^. Furthermore, dysregulation of the *ADAM12* gene is implicated in pathogenesis of pre-eclampsia in pregnant women^25^. This gene is expressed in the mare^26^, and the expression is similar at 1.5, 4, 6 and 10 months of gestation^27^. It is unknown if the level of expression changes in high-risk equine pregnancies or how LNPEP reaches the maternal circulation. The amino acid sequence Phe^154^Ala^155^, which is the cleavage site for the release of placental LNPEP in maternal circulation is restricted to members of the Homindae family, and the horse lacks sequence alignment with the region of LNPEP containing the cleavage site based on the human amino acid sequence^28^. Therefore, little difference between serum LNPEP in pregnant and non-pregnant mares would be expected and is consistent with similar levels of LNPEP in the blood of pregnant and non-pregnant mice^28,29^.

Very little research has been done to characterize equine LNPEP despite the central role of oxytocin in reproduction and its functional relationship to oxytocin. The endometrial flush fluid of non-pregnant mares at Day 4, 8, 12, 14, 16, 18 and 20 post-ovulation had LNPEP activity, which peaked at Day 12^30^. Those authors suggested that the LNPEP was most likely derived from serum transudation^30^, but no studies were performed to identify if the equine endometrium expressed LNPEP. Oxytocinase, with a molecular weight of 115 kDa, was identified in full term equine placental extracts^31^. Additionally, a study in 40 pregnant and 10 non-pregnant mares that measured monthly changes in the circulating levels of LNPEP in plasma via a colorimetric test did not find any differences between pregnant and non-pregnant mares^32^. This suggested that LNPEP levels are not related to increases in placental mass associated with the stage of gestation in mares, therefore, placental LNPEP in the horse may not be a major contributor to the circulating plasma level. Further studies using ELISA and molecular techniques are indicated to confirm those findings.

In sheep, LNPEP has a widespread distribution in the reproductive tract, with the myometrial outer layer and endometrial luminal epithelial having the highest level of LNPEP. However, when exogenous estrogen was administered to ovariectomized sheep, a significant decrease in LNPEP was identified in the outer myometrial layer^33^, suggesting that estrogens may regulate LNPEP activity. In human endometrium, LNPEP expression is localized to the epithelial cells^34^. Furthermore, progesterone decreases LNPEP in an in vitro model, increased the sensitivity to oxytocin and increased the secretion of prostaglandin E2 (PGE2)^35^. Both aforementioned studies have suggested that LNPEP has a role in human embryonic implantation.

There is a paucity of information on the presence of LNPEP in different tissues of mares and conflicting information regarding the expression of oxytocin in the corpus luteum. It is unknown if LNPEP changes during the expected time of maternal recognition of pregnancy (MRP), late gestation and parturition. We hypothesized that LNPEP in mares would be similar to other non-Homidae species and would be found widely distributed in tissues and serum. Our second hypothesis was that OXT will be expressed in the luteal tissue but have fundamentally different tissue characteristics compared to ruminant species. Therefore, our objectives were to (1) characterize serum LNPEP in mares during the estrous cycle, early pregnancy, late pregnancy and post-partum; (2) to characterize LNPEP and OXT in placental tissues of mares; (3) to investigate the tissue distribution and characterize LNPEP and OXT in reproductive and other tissues of non-pregnant mares.

## Results

### Experiment 1 serum LNPEP and progesterone levels in pregnant and non-pregnant mares

**Figure 1:**
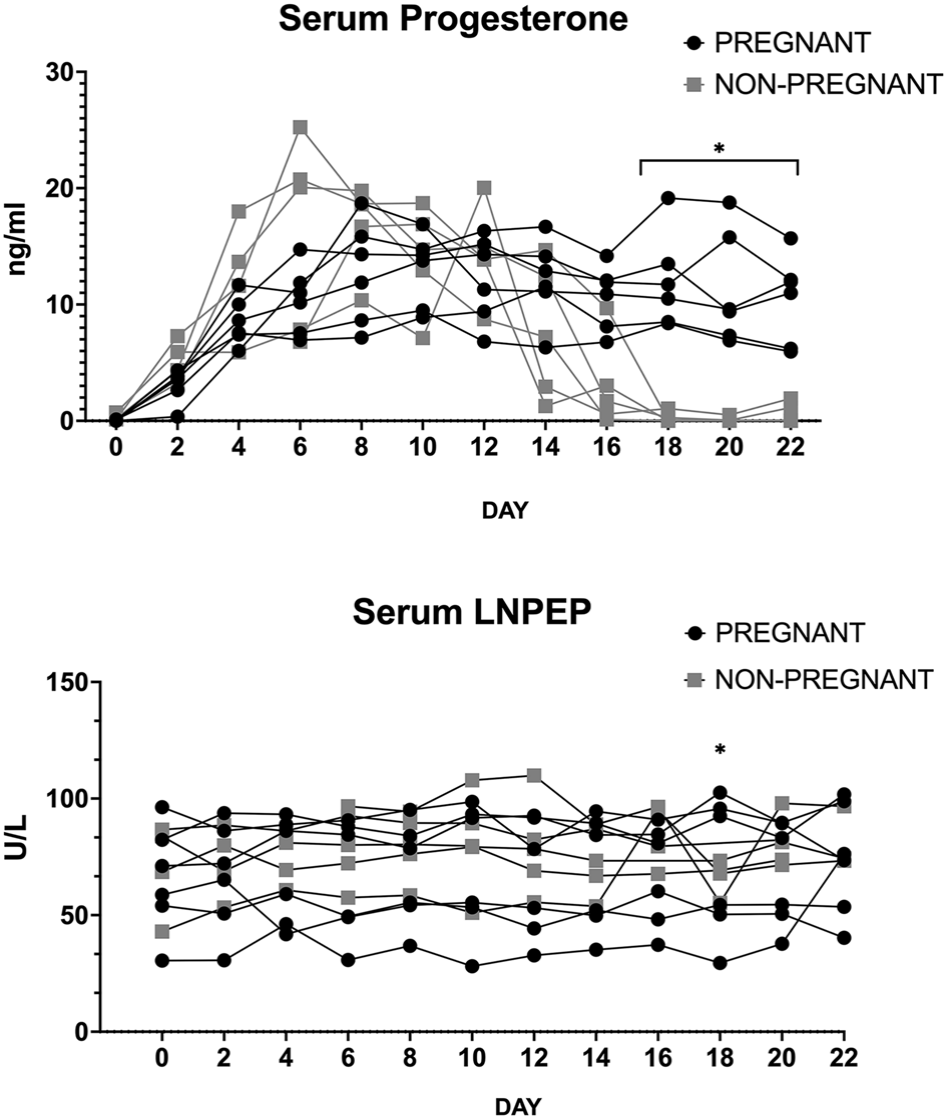
Serum progesterone (ng/ml) (upper panel) and oxytocinase (LNPEP) (U/L) (lower panel) of each individual animal in non-pregnant (n = 5) (grey) and pregnant mares (n = 6) (black) from the Day of ovulation (0) to 22. Differences (*p* ≤ 0.05) at the various time points between groups are indicated by stars.

The main outcome of the experiment was a comparison of serum progesterone and LNPEP levels through one estrous cycle in non-pregnant mares with the levels from mares in the first 22 days of pregnancy. Figure 1 demonstrates the graph for serum progesterone and LNPEP in non-pregnant (n = 5) and pregnant mares (n = 6) by Day (D). Serum progesterone was different between pregnant and non-pregnant mares (*P* ≤ 0.001) with a median and interquartile range (IQR) of 6.44 (0.39, 14.23) in non-pregnant mares and 10.98 (9, 12.19) in pregnant mares. There was a significant difference between the two groups staring at D18 (*P* ≤ 0.05).

No difference between serum LNPEP (U/L) between groups of pregnant and non-pregnant mares was identified and median and interquartile range (IQR) LNPEP concentration (U/L) in non-pregnant mares was 79.43 (73.01, 81.43) and in pregnant mares was 67.45 (65.75, 69.86). However, D18 non-pregnant mares had decreased LNPEP concentration (U/L) 68.6 (58.4, 72.3) compared to pregnant mares 73.4 (45.1, 97.4) (*P* ≤ 0.01). In the data analysis, the progesterone levels did not have an effect on the LNPEP concentration (*P* = 0.3), but there was a mare effect (*P* ≤ 0.04) on serum LNPEP.

### Experiment 2 Peripartum and postpartum serum LNPEP levels and characterization of placental LNPEP and OXT

**Figure 2:**
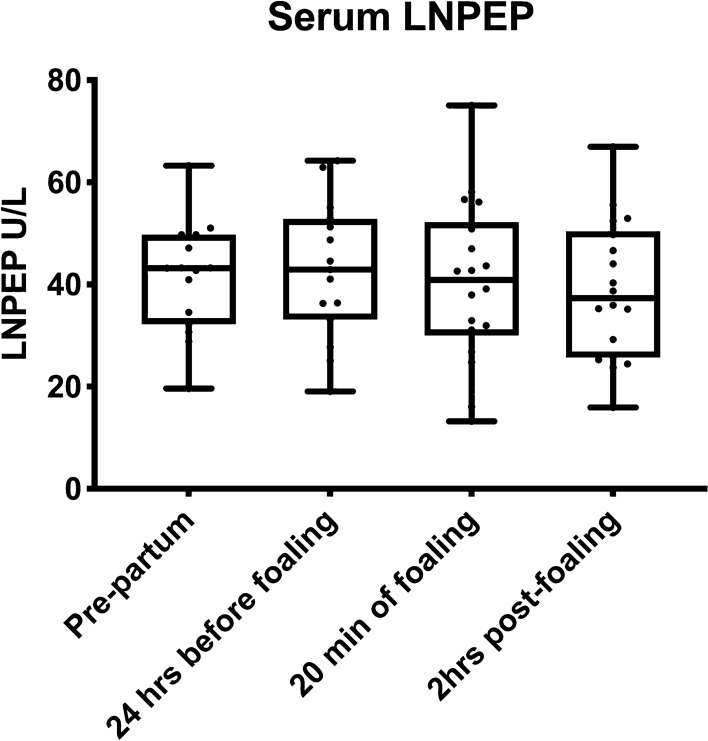
Median and interquartile (IQR) oxytocinase (LNPEP) (U/L) in mare serum (n = 18) pre-partum (around 320 days of gestation), 24 h before foaling, within 20 min of foaling and 2 h post-foaling.

**Figure 3:**
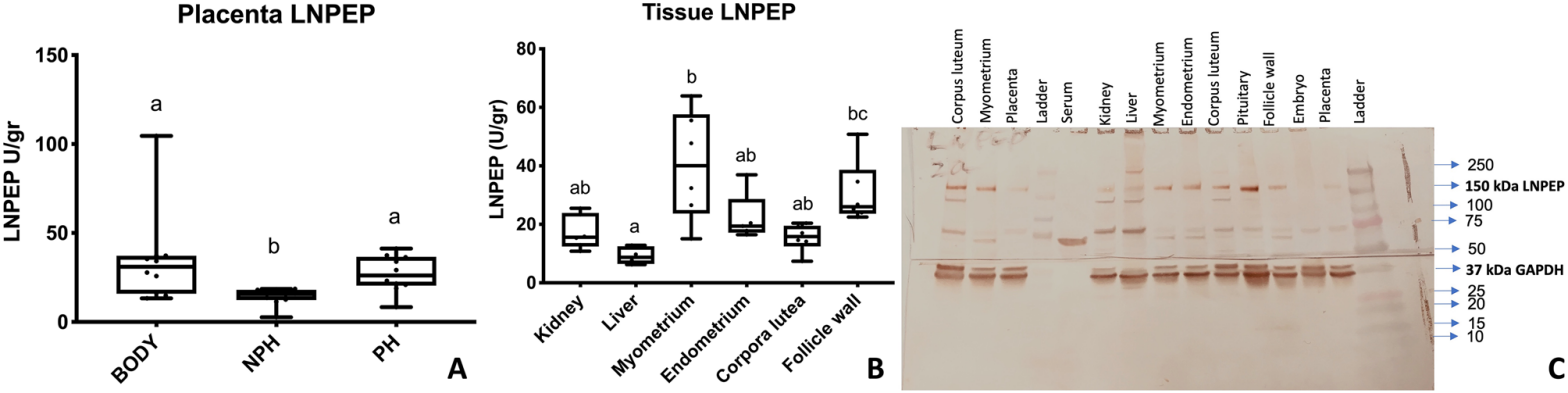
Median and interquartile (IQR) of oxytocinase (LNPEP) U/gr protein in: Panel A Body, Non-pregnant horn (NPH) and Pregnant horn (PH) of equine placenta (n = 7); Panel B LNPEP (U/gr) in different tissues from (n = 6) mares; and Panel C is a western blot performed using rabbit anti- LNPEP at 1:1000 (150 kDa) on the top portion of the gel and mouse anti-GAPDH (37 kDa) at 1:1000 on the bottom portion of the gel. Lane labels of corresponding tissues. Significance differences (*p* ≤ 0.05) between tissues, and placental locations are indicated by different superscripted letters.

The main outcomes of this experiment were: a comparison of serum LNPEP levels in the peripartum period of mares (n = 18); characterization of placental (n = 7) LNPEP using ELISA, immunostaining, qPCR, western blot, and LC–MS/MS; and characterization of placental OXT using immunostaining, qPCR, western blot, and LC–MS/MS. The mean gestational age of the mares (n = 18) at foaling was 343 ± 7.1 days (range 332–361 days). There was no significant effect of day/hour on serum LNPEP levels. The median serum LNPEP concentration (U/L) and interquartile range (IQR) at sampling times were: pre-partum 43.2 (32.2, 49.8), 24 h to foaling 42.9 (33.1, 52.8), within 20 min after foaling 40.87 (30, 52.2), and 2 h post-foaling 37.3 (25.7, 50.4) (Fig. 2). There was a significant effect of placental region on LNPEP (*P* = 0.006), with body and pregnant horn (*P* = 0.01 and *P* = 0.001, respectively) having significantly higher levels than non-pregnant horn. The LNPEP concentration U/mg (median [IQR]) by placental region were: B (31 [15.6, 37.2]), NPH (15.75 [12.3, 18.06]), and PH (26.11 [20.38, 36.58]) (Fig. 3A). Supplementary Fig. 1 (Page S1, Image 1A) is a gel prepared from tissue extracts including kidney, liver, ovary, CL, myometrium, endometrium, embryo, and placenta and demonstrates a band corresponding to LNPEP at about 150 kDa for each tissue. The initial two immunoblots of freshly prepared tissue demonstrated a band for the placenta for LNPEP for the expected molecular weight (150 kDa) (Supplementary Fig. 1, Page S1, S6 and S7). However, the later WB, the placental band was not visible (see 150 kDa region of the WB in Supplementary Fig. 1, page S8–10).

**Figure 4:**
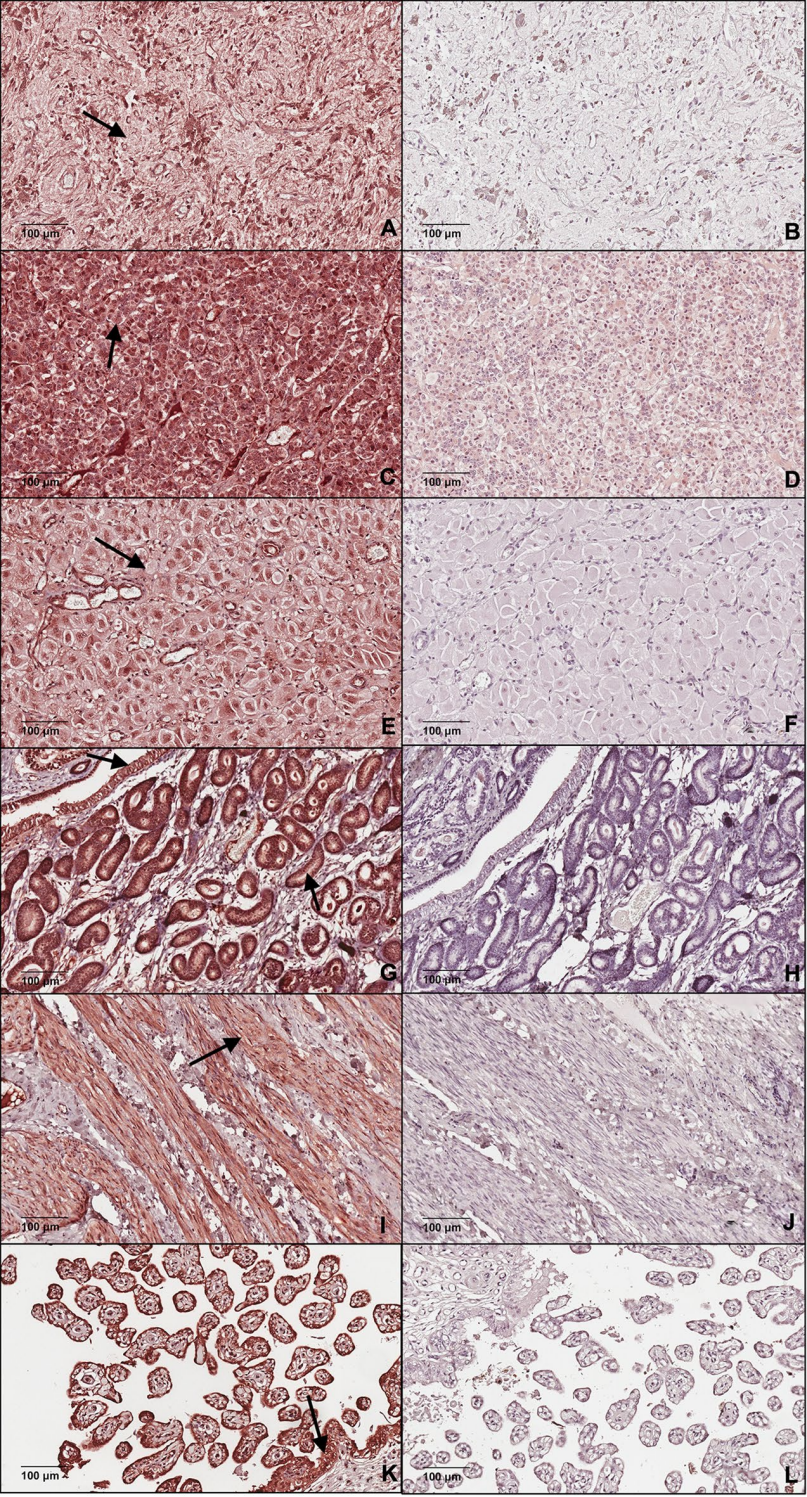
Photomicrograph demonstrating immunolocalization of LNPEP in: (**A**) posterior hypophysis, (**C**) anterior hypophysis, (**E**) corpus luteum, (**G**) endometrium, (**I**) myometrium and (**K**) placenta. Black arrow shows cytoplasmatic specific staining. Negative epitope controls for each tissue (**B**, **D**, **F**, **H**, **J** and **L**).

**Figure 5:**
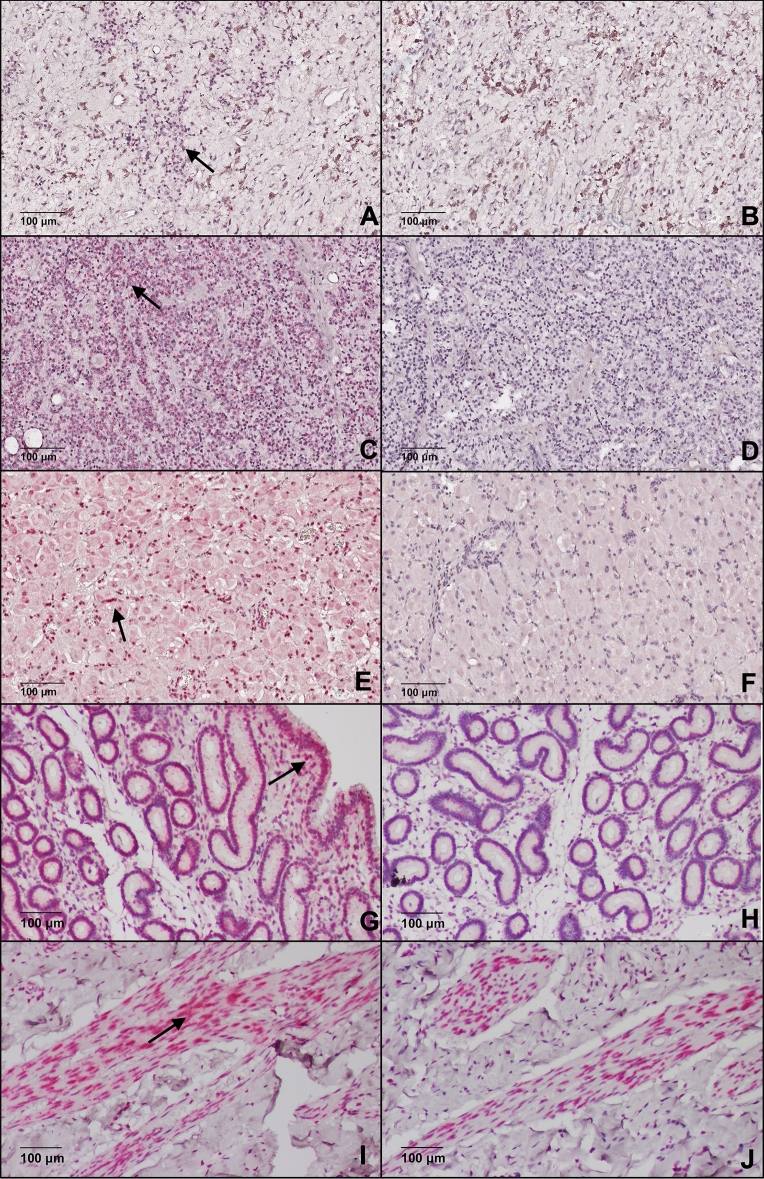
Photomicrograph demonstrating mRNA expression of LNPEP (pink dots) in: (**A**) posterior hypophysis, (**C**) anterior hypophysis, (**E**) corpus luteum, (**G**) endometrium and (**I**) myometrium. Black arrow shows specific staining. Control DAB for each tissue (**B**, **D**, **F**, **H** and **J**).

No band was detected for OXT at the expected molecular weight (10 kDa) in placental tissues (see 10–20 kDa region of WB of Supplementary Fig. 1, page S2 and S4). The portions of the Coomassie blue gel corresponding to the molecular weight regions of the placental tissue LNPEP and OXT was processed by LC–MS/MS and contained both LNPEP and OXT despite the lack of detectable bands on WB (Tables 1 and 2). Evaluation of IHC showed positive diffuse immunostaining in the lining cells of the allantois and amnion, smooth muscle cells of arteries and of the endothelial cells of arteries; and there was positive diffuse cytoplasmic staining of the trophoblast for LNPEP, with no staining in the negative control (Fig. 4). For OXT, some smooth muscle cells of blood vessels were noted to have diffuse cytoplasmic staining, and there was no staining of the negative epitope control (Fig. 5).

**Table 1:** Identification of the leucyl-cystinyl aminopeptidase (LNPEP) by the liquid chromatography-tandem mass spectrometry (LC–MS/MS) of the 150 kDa bands of various tissues from the Coomassie blue gel.

Tissue	Protein FDR Conf	Accession	Description	# Peptides	# PSMs	# Unique Peptides	# AAs	MW [kDa]	Found in sample
Embryo	High	1333575043	leucyl-cystinyl aminopeptidase isoform X2 [Equus caballus]	12	14	12	1026	117.3	High
Placenta	High	1333575043	leucyl-cystinyl aminopeptidase isoform X2 [Equus caballus]	10	10	10	1026	117.3	High
Pituitary	High	1333575043	leucyl-cystinyl aminopeptidase isoform X2 [Equus caballus]	12	15	12	1026	117.3	High
Combined 15 kDa bands from the Coomassie blue gel of: CL, endometrium, myometrium	High	1333575043	leucyl-cystinyl aminopeptidase isoform X2 [Equus caballus]	6	9	6	1026	117.3	High

Protein false discovery rate confidence (Protein FDR Conf) = High means FDR < 0.01; number (#) of peptides = the number of distinct peptide sequences in the protein group; Number of peptide spectrum matches (#PSMs) = the total number of identified peptide sequences for the protein; # Unique peptide = number of the peptide sequences unique to a protein group; Number of amino acids (#AA) = the sequence length of a protein; Molecular weight (MW[kDa]) = calculated molecular weight of the protein; Found in sample = high, peak found, or peak not found.

**Table 2:** Identification of neurophysin-oxytocin (OXT) by the liquid chromatography-tandem mass spectrometry (LC–MS/MS) from the 10-15 kDa bands of various tissues from the Coomassie blue gel.

Tissue	Protein FDR Conf	Accession	Description	# Peptides	# PSMs	# Unique Peptides	# AAs	MW [kDa]	Found in sample
Corpus luteum	High	1333604671	Low quality protein: oxytocin-neurophysin 1 [Equus caballus]	2	5	2	162	17.1	High
Placenta	High	1333604671	Low quality protein: oxytocin-neurophysin 1 [Equus caballus]	1	2	1	162	17.1	High
Pituitary	High	1333604671	Low quality protein: oxytocin-neurophysin 1 [Equus caballus]	5	90	4	162	17.1	High
	High	128070	RecName: Full = Oxytocin-neurophysin 1; Short = OT-NPI; Contains: RecName: Full = Oxytocin; AltName: Full = Ocytocin; Contains: RecName: Full = Neurophysin 1; Flags: Precursor	5	85	1	105	10.7	High
	High	1333604673	oxytocin-neurophysin 1 [Equus caballus]	5	76	1	125	12.7	High

Protein false discovery rate confidence (Protein FDR Conf) = High means FDR < 0.01; number (#) of peptides = the number of distinct peptide sequences in the protein group; Number of peptide spectrum matches (#PSMs) = the total number of identified peptide sequences for the protein; # Unique peptide = number of the peptide sequences unique to a protein group; Number of amino acids (#AA) = the sequence length of a protein; Molecular weight (MW[kDa]) = calculated molecular weight of the protein; Found in sample = high, peak found, or peak not found.

**Figure 6:**
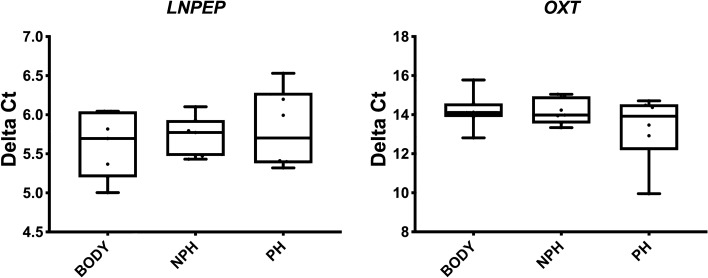
Relative abundance (Delta Ct) of *LNPEP* and *OXT* in the equine placenta Body, Non-pregnant horn (NPH) and Pregnant horn (PH). Delta Ct concentrations have an inverse relationship to mRNA abundance.

There was no difference in the relative abundance of *LNPEP* and *OXT* between placental regions (*P* = 0.64 and *P* = 0.82, respectively), and *OXT* had low and inconsistent levels of expression in the placenta overall (Fig. 6).

### Experiment 3 characterization of LNPEP and OXT in reproductive tissues

Liver, kidney and reproductive tissues from 6 non-pregnant mares were collected. Outcomes of this experiment included characterization of LNPEP and OXT using ELISA, western blot, LC-MS/MS, immunostaining, qPCR and RNA in situ hybridization. Regarding tissue LNPEP levels as measured by ELISA, the tissues ranked from highest to lowest concentration (median tissue units per 100 mg of protein, and IQR) were: myometrium 40.1 (23.7–57.6), follicle wall 26 (23.6–38.6), endometrium 19.4 (17.1–28.7), kidney 15.6 (12.4–23.9), corpus luteum 15.8 (12.4–19.5), and liver 8.7 (6.5–12.5) (Fig. 3 B). The WB showed a band around 150 kDa for the LNPEP for all tissues, except placenta. The GAPDH 37 kDa band demonstrated evenly loaded protein for all the tissues alongside the LNPEP (Fig. 3C). For OXT, a band around 10 kDa was identified for pituitary and a faint band was visualized for the CL, but not for endometrium or myometrium (Supplementary Fig. 1, Page S2). The LC-MS/MS confirmed the identity of LNPEP in the bands submitted for all the tissues (Table 1), and OXT was correctly identified in equine pituitary and CL (Table 2), but not in equine endometrium and myometrium. Supplementary Fig. 1 shows the summary of the most representative uncropped western blot and Coomassie blue gels for LNPEP and OXT.

Immunohistochemistry results demonstrated strong immunostaining for LNPEP in all the reproductive tissues. None of the negative epitope control tissues had any immunostaining. The positive control for LNPEP was pituitary where immunostaining in the adenohypophysis was in serum in blood vessels, and the cytoplasm of interstitial cells, and of cells of the neurohypophysis. In the endometrium, there was immunostaining of the serum within blood vessels, the epithelial cells had intense diffuse cytoplasmic and nuclear immunostaining, and endometrial stromal cells had positive cytoplasmic immunostaining. The myometrial smooth muscle cells had cytoplasmic staining and there was immunostaining of some nuclei. There was immunostaining of serum in vessels, and cytoplasmic immunostaining of luteal cells of the corpus luteum (Fig. 4).

The LNPEP RNAscope demonstrated expression in the adenohypophysis and neurohypophysis. There was frequent staining of cytoplasm particularly near nuclei of endometrial epithelial cells, and much more so than in controls for the endometrium. Staining of endometrial stromal cell nuclei was visible throughout along with staining of the nuclei of smooth muscle cells. There was abundant stippled staining in smooth muscle nuclei of the myometrium. The CL had staining of nuclei of luteal cells. There was no signal in the negative control sections of the CL and pituitary; the only signal in the uterus was some positive cytoplasmic signal near the nucleus of about 10% of the endometrial epithelial cells and in approximately 50% of the nuclei of smooth muscle cells, so this was considered background (Fig. 5).

**Figure 7:**
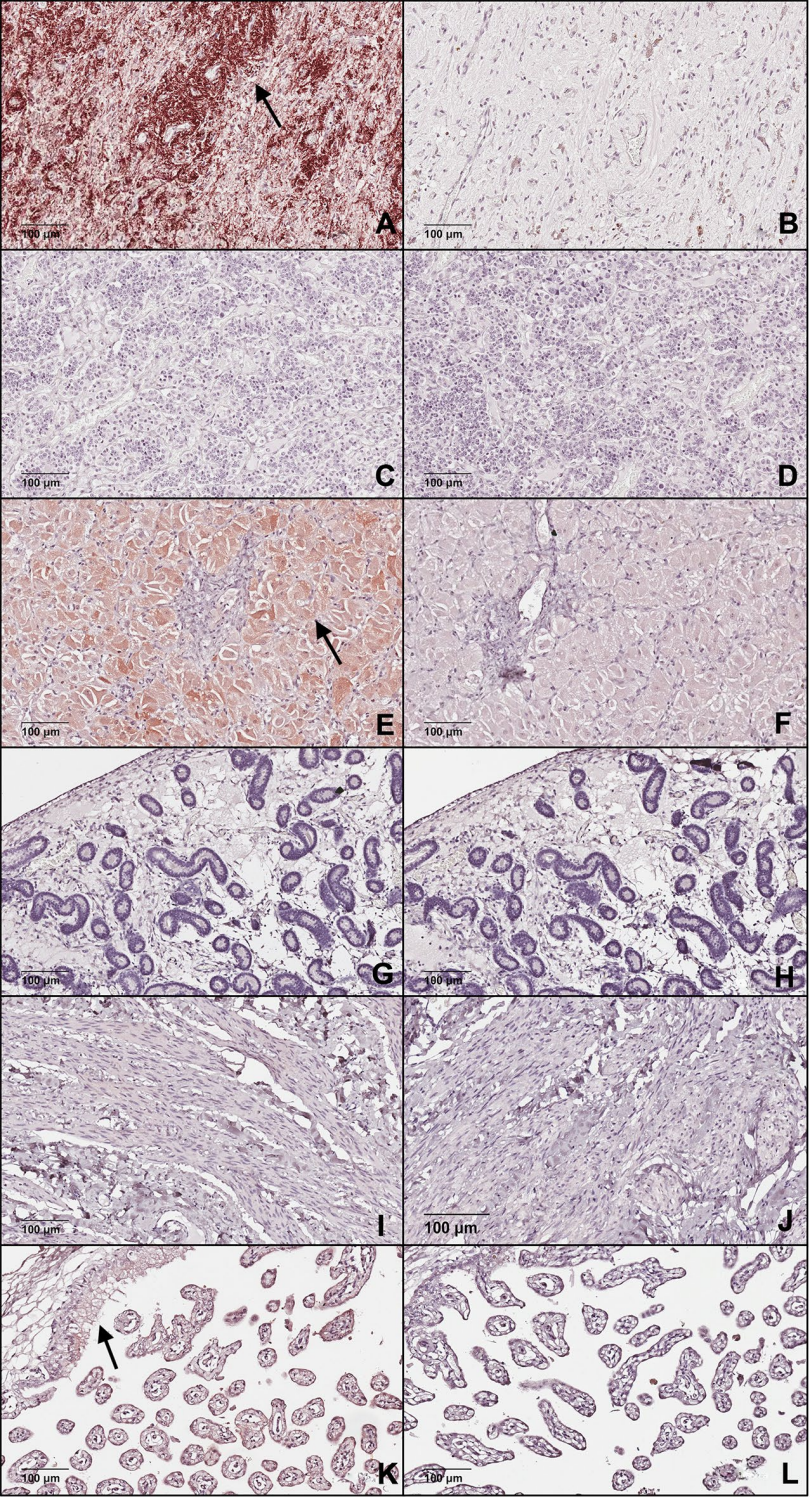
Photomicrograph demonstrating immunolocalization of OXT in: (**A**) neurohypophysis, (**E**) corpus luteum, and (**K**) the placenta and negative staining uptake for OXT is shown in the (**C**) adenohypophysis, (**G**) endometrium and (**I**) myometrium. Black arrow shows specific staining. Negative epitope controls for each tissue (**B**, **D**, **F**, **H**, **J** and **L**).

**Figure 8:**
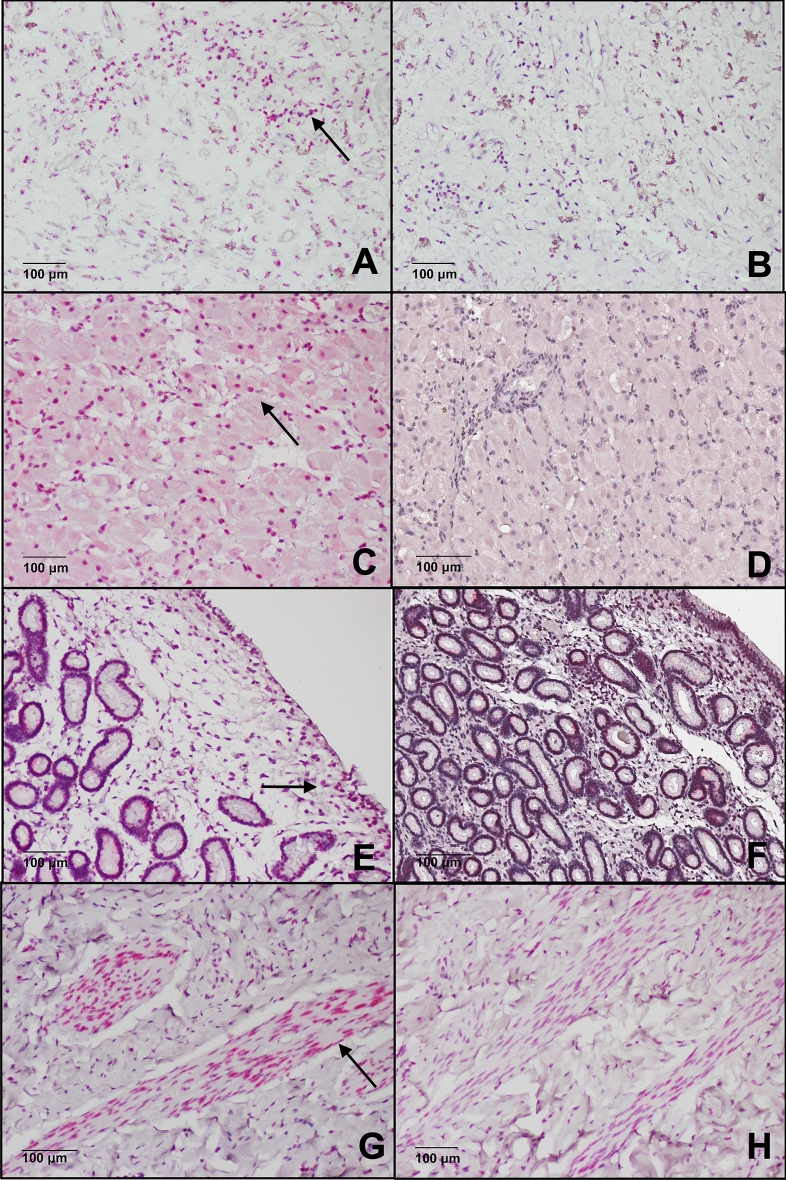
Photomicrograph demonstrating mRNA expression of OXT (pink dots) in: (**A**) posterior hypophysis, (**C**) corpus luteum, endometrium (**E**) and myometrium (**H**). Black arrow shows specific staining. Control DAB for each tissue (**B**, **D**, **F** and **H**).

The OXT immunohistochemistry demonstrated positive cytoplasmic staining in the neurohypophysis and no immunostaining was detected in the adenohypophysis. There was diffuse cytoplasmic immunostaining of the luteal cells of the corpus luteum. No immunostaining was noted in the endometrium or myometrium. There was no immunostaining in the negative control tissues (Fig. 7). The RNAscope demonstrated OXT in the neurohypophysis. The CL had signal on the nuclei of endocrine luteal cells. There was some staining of the cytoplasm of the endometrial epithelial cells. There was a positive signal of the nuclei of smooth muscle cells of blood vessels and the myometrium. The negative control sections of the pituitary and CL were negative, but endometrium and myometrium had some positive signal in the cytoplasm near the nucleus of low numbers of the endometrial epithelial cells, so this was considered background (Fig. 8).

## Discussion

The first hypothesis that LNPEP would have a serum pattern with less variation than Hominidae species during various reproductive states was upheld. The serum concentration of LNPEP was not different between non-pregnant and pregnant mares during early pregnancy (D0–D22), and between late gestation and the early post-partum period. We compared the serum LNPEP concentrations of early pregnant mares (D0–22) 67.5 (65.4, 74.1) to the pre-partum mare 43.7 (19.7, 63.2), the latter has significantly decreased concentrations of LNPEP (*P* = 0.0001). This finding is in agreement with previous study that evaluated monthly blood samples from both non-pregnant and pregnant animals and found similar LNPEP levels^36^. Placental LNPEP is unlikely to contribute to serum LNPEP as LNPEP concentration does not increase as placental mass increased nor did it decrease dramatically post-partum. The basis for this finding is that the cleavage site for releasing the membrane bound form of the LNPEP from the placenta into the soluble form in blood depends on the human amino acid sequence (Phe^154^Ala^155^), which is not present in horses due to a lack of sequence alignment^28^. Therefore, placental LNPEP may not be cleaved for release into the circulation and it may not be a useful serum biomarker for pre-term delivery and other high-risk pregnancy associated problems in the equine, as it is in women^22,23^. There was an individual mare effect on the serum LNPEP concentration. The increased plasma volume of pregnant mares compared to non-pregnant counterparts explains the lower level in late pregnancy^37^, but we did not attempt to measure plasma volume in our study.

We investigated the association between serum progesterone in LNPEP concentrations. Serum progesterone concentration started to diverge between pregnant mares and non-pregnant from D14–16, which corresponds with luteolysis^36^. A statistically significant difference in progesterone was noted from D18 on, where pregnant mares maintained an elevated progesterone concentration and the non-pregnant groups had undergone luteolysis. Changes in progesterone concentration did not affect serum LNPEP levels. Based on the lack of a mechanistic experiment, this observation could be due to chance. However, Diel de Amorim et al.^52^ reported the effect of various treatments and physiologic states (carbetocin, meclofenamic acid, putative luteal prolongation with oxytocin during diestrus and pregnancy) on levels of serum progesterone and LNPEP from D12–15 after ovulation. The authors in that study identified significant group differences between LNPEP concentrations in the time frame studied, but the differences were not statistically related to changes in serum progesterone levels. This data suggested progesterone may not regulate serum LNPEP levels^38^. However, in an in vitro model using Ishikawa cells, a cell line derived from human endometrial adenocarcinoma cells, LNPEP secretion was decreased by progesterone treatment. The decrease in LNPEP increased the sensitivity of this cells to OXT, which then resulted in an increased secretion of PGE2^35^, which is luteotrophic. The luteotrophic action of PGE was shown by direct injection of luteal tissue with PGE and high dose intrauterine administration of PGE^39^. It is unknown if progesterone has an effect on LNPEP at the tissue level, and therefore promotes PGE2 secretion for luteal maintenance during early pregnancy. It is likely that LNPEP may tightly regulate OXT in endocrine luteal tissue and therefore further studies are required to understand the role of LNPEP and OXT on luteal regulation.

Serum LNPEP is expected to be involved in rapid clearance of oxytocin released by the pituitary as tight regulation of this hormone is needed. The presence of varying amounts of tissue LNPEP may explain the different physiologic effects of oxytocin, such as the capacity to induce luteostasis, based on the dose, route, duration, and frequency when administered. In ruminant species, administration of oxytocin in the early post ovulatory period results in an early return to estrus as it interferes with the formation of the corpus luteum. In light horse mares 60 IU of oxytocin, administered intramuscularly (IM) from days 7–14 of the estrus cycle, results in luteostasis in a large proportion of mares^8,40^. Low dose (10 IU) intravenous (IV) administration of oxytocin did not prolong the luteal phase while IM administration did, a result which may be explained by the more rapid clearance of oxytocin by serum LNPEP when administered IV. Lactating mares experience many episodes per day of oxytocin release in response to suckling and do not experience prolonged luteal phases. In our experiment the concentration of LNPEP was measured and not the specific activity of the LNPEP. There may be differences in specific activity of the LNPEP just as there are differences in tissue and serum concentrations. Further study is needed to determine if these differences exist and additionally to examine the effect of daily oxytocin treatment on serum and luteal LNPEP concentrations and expression. Oxytocin administration may induce changes in luteal tissue that involve LNPEP and facilitate luteostasis.

We previously reported on the endometrial and luteal *LNPEP* abundance in diestrus and pregnant mares during the expected time of pregnancy recognition. We found that D12 diestrus mares had a higher endometrial abundance than D8 or D15 pregnant and non-pregnant endometrium. In luteal tissue, *LNPEP* abundance was low on D8 then increased in both groups on D10 and D12 and remained high on D15 diestrus.^41^ Consistent with the presence of endometrial and luteal *LNPEP*, we reported that LNPEP is present in a wide variety of tissues including the equine placenta. There was a difference in placental LNPEP with the body and pregnant horn having higher levels than the non-pregnant horn. Our findings are consistent with a study that reported the molecular weight of LNPEP as 115 kDa in an equine placental extract^31^, and a recent transcriptomic study has demonstrated the expression of LNPEP in the equine placenta throughout gestation^42^. In our other tissues the highest concentration of LNPEP per gram of protein was present in the myometrium and placenta, which is similar to other species^16,18,19^.

Oxytocinase is also known to be rapidly degraded due to the endogenous proteases that are released post-partum and involved in placental separation, and which would be present during storage of the tissue extract^43^. The placental tissue in this research was naturally delivered and samples were collected post-partum. We attribute the effect of endogenous proteases as to why we did not consistently identify a band for LNPEP in our later WB, as the the degraded LNPEP would not be identified in the later gels. However, we were still able to identify both LNPEP and OXT by more sensitive methods such as LC-MS/MS analyzing the portions of the Comassie blue gel corresponding to their molecular weights.

Equine endometrial and luteal *OXT* in early pregnancy has been reported showing changes in abundance based on day post ovulation and pregnancy status^41^. The highest *OXT* endometrial abundance was reported on day 10 where pregnant mares had greater level than diestrus or pregnant mares on D8 and D15. In luteal tissue increased *OXT* abundance was found on D10 and D12 with pregnant mares having higher levels than diestrus mares. Oxytocin is reported to be challenging to measure due to factors related to sample preparation and the different states in which the oxytocin molecule exists. Oxytocin is present as: a preprohormone, oxytocin neurophysin-1, a 9 amino acid peptide, or as a ligand bound to oxytocin receptor (OTR)^44^.

In our study, we were able to immunolocalize OXT in the neurohypophysis and CL and detect OXT by LC-MS/MS in the pituitary, placenta, and corpus luteum but not in the endometrium. The primary antibody utilized showed no cross reactivity with neurophysin-1 and was chosen to detect the biologically active free hormone. The lack of endometrial OXT could arise because the samples in this study were obtained during diestrus in the mares, when the quantity of OXT in those tissues was low, compared with estrus where a higher staining intensity has been reported^5^. There may have been fast degradation of OXT during the protein extraction process which may have prevented detection and additionally the antibody may not recognize oxytocin bound to either neurophysin-1 or its receptor. Our findings are in contrast to a previously published research study^6^. The authors demonstrated oxytocin immunostaining in the small luteal cells of the midcycle CL in mares, which they suggest may represent oxytocin bound to receptors, as there was no immunostaining for neurophysin. We demonstrated the presence of OXT in the equine CL consistently by different molecular tools. Oxytocin was immunolocalized to the large endocrine luteal cells, which is in accordance with published studies in ovine endocrine luteal cells^45^. Oxytocin signal was detected by RNAscope in the nuclei of CL and myometrial smooth muscle cells. The perinuclear and nuclear immunostaining and localization of oxytocin using RNAscope may be due to background or cross-reaction with matrix or potentially detection of the internalized oxytocin OTR complexes and nuclear relocalization as reported by Kinsey et al.^46^. The OTR is a transmembrane heptahelical G-protein coupled receptor^46^. Kinsey et al.^46^ described the constitutive and ligand-induced nuclear localization of ligand bound OTR in normal human fibroblasts and neoplastic cells. The nuclear localization in the fibroblast cells was dependent on OTR ligand binding. Binding of OTR may occur by locally produced or systemically secreted oxytocin. The bound OTR internalizes and is found in vesicles that accumulate in the perinuclear area, and some of the bound OTR enters the nucleus. Confocal imaging studies showed that oxytocin treated cells had strong immunolabeling of the endoplasmatic reticulum associated with the outer nuclear envelop as well as antibody positive material in the nucleus^46^. Similar in vitro studies of equine luteal cells would have to be performed to determine if ligand bound OTR complexes enter the nucleus. There is a need to address the biologic function of OTR and ligand bound OTR in cell nuclei. These findings suggest that OXT in the equine CL may have a paracrine or autocrine role during luteolysis or luteostasis, contrary to the reports that the equine CL is not a source of OXT^6^. This is quite distinct from physiologic regulation of the ruminant CL where OXT is packaged in much higher amounts in granules of large luteal cells. Luteal OXT is released systemically through exocytosis during luteolysis, which causes an increase in endometrial prostaglandin F release^47^. We accepted our second hypothesis that OXT would be present in equine luteal tissue but have fundamentally different characteristics compared to ruminant species. Further research should be undertaken to determine the role of equine luteal OXT in luteolysis and pregnancy maintenance.

A limitation of this study is the lack of measurement of oxytocin and prostaglandin concentrations in serum and peripheral tissues, as this was beyond the scope of this study. Tissues that produce or store OXT such as the neurohypophysis and the corpus luteum have both OXT and LNPEP, suggesting that LNPEP has important regulatory functions prior to and after OXT secretion.

## Conclusion

Oxytocinase is likely a main regulator of OXT concentrations in mares and acts systemically through serum LNPEP and locally through tissue LNPEPs. The serum concentration of LNPEP did not vary in the first 21 days post ovulation between pregnant and non-pregnant mares and was not affected by progesterone concentrations. Placental LNPEP does not contribute to the circulating pool of LNPEP and is unlikely to be a useful biomarker for high-risk pregnancies in the mare. Oxytocinase is present in varying concentrations in reproductive and other tissues.

Equine neurohypophysis, luteal and placental tissues are sources of OXT, although in luteal tissues the expression and protein levels of OXT is low, which suggests that oxytocin has a paracrine or autocrine role during luteal regression. The features of equine luteal OXT are distinctively different from ruminant luteal tissue.

## Materials and methods

### Animals

In total thrity-five healthy light breed mares ranging in age from 3 to 16 years old, with a mean and standard deviation (± SD) age of 8.8 ± 3.5 years were used in this study. All horses were fed forage, concentrate, water and trace mineral supplementation to meet their requirements. All methods were carried out in accordance with relevant guidelines and regulations, and the entire study was approved by the Ethical Committee of the University of Saskatchewan (UCACS protocol # 2012004) and the University of Prince Edward Island (16–001 and 6,006,500). The researchers followed the recommendation of the ARRIVE guidelines.

### Experiment 1 Serum LNPEP and progesterone levels in pregnant and non-pregnant mares

Eleven mares were used in this experiment, consisting of 5 non-pregnant (control) and 6 pregnant mares. Transrectal palpation and ultrasound were used to determine normal cyclicity prior to enrolment of the animals in the study. Daily reproductive ultrasound examinations were performed once estrus was detected (≥ 30 mm with at least a grade 1 uterine edema, soft uterus and no CL present) until the day of ovulation (D0). Mares that were assigned to the pregnant mare groups were hand bred by a single fertile stallion every other day until ovulation was detected. Pregnancy was confirmed by transrectal ultrasound at 12, 14- and 21-days post-ovulation to determine pregnancy status and fetal viability. Jugular blood was obtained every other day from D0 until D21 in pregnant mares and until the following ovulation in non-bred mares (around 21–22 days) for measurement of LNPEP and progesterone (P4). Blood samples were refrigerated, centrifuged and the serum was stored frozen in aliquots at − 20 °C until the time of analysis.

### Experiment 2 Peripartum and postpartum LNPEP levels and characterization of placental LNPEP and OXT

Eighteen late pregnant Standardbred mares were used in this experiment. The reproductive history was collected on each mare, including last breeding date and confirmation of the mare’s 14-day and 30-day pregnancy checks. Owner consent form was acquired for all mares enrolled in the study and sample collection procedures adhered to the high standard of veterinary care. Daily monitoring of physical changes (tail head laxity, sacrosciatic ligament, vulvar elongation) and udder development were observed from 320 days of gestation until parturition. Pre-foaling mammary secretion was monitored daily in late afternoon for electrolytes and pH to determine foaling date as previously described^48^. All foalings were observed. Jugular blood collection was performed: prepartum (320–336 days of gestation), 24 h before parturition, 20 min and 2 h after foaling. Blood was processed as above. Serum was stored frozen at − 20 °C until analysis. Immediately following parturition, the chorioallantoic membrane tissues were retrieved from naturally delivered placentae from the body (B), pregnant horn (PH), and non-pregnant horn (NPH) in eight (n = 8) mares, then divided into two pieces: one piece was stored frozen at − 80 °C until analysis, and the second was placed in 10% formalin for 24 h and paraffin embedded for immunohistochemistry (IHC).

### Experiment 3 characterization of LNPEP and OXT in reproductive tissues

Samples of the reproductive trat and other tissues were collected from six healthy mares that were used as control animals in another study at post-mortem examination. Euthanasia was carried out via jugular vein injection with an overdose of pentobarbital sodium. The reproductive tract was examined to determine follicular activity and stage of the estrous cycle. Mares were determined to be in diestrus based on medium size follicles, lack of endometrial edema and a presence of a mature corpus luteum (CL). Tissue samples (liver, kidney, myometrium, endometrium, corpus luteum, follicle wall and pituitary) were collected at necropsy. Tissues were immediately placed into a cryovial, snap frozen in liquid nitrogen and stored at − 80 °C. Portions of the reproductive tissues (myometrium, endometrium, CL, ovary and pituitary) were placed in 10% formalin for 24 h and paraffin embedded for IHC and in situ hybridization (RNAscope).

### Serum progesterone and oxytocinase

All serum samples from animals in Experiment 1 were analyzed for progesterone and LNPEP. The serum from mares in Experiment 2 were only analyzed for LNPEP. Serum progesterone analyses from the mares was performed at the Endocrinology Laboratory at the Western College of Veterinary Medicine in a single radioimmunoassay (ImmuChem™ Coated Tube Progesterone 125 RIA Kit, ICN Pharmaceuticals, Costa Mesa, CA). Declining daily progesterone levels along with levels below 2 ng/mL progesterone were considered consistent with luteolysis.

A second aliquot of serum was used to measure the changes in circulating LNPEP levels using a commercially available enzyme-linked immunosorbent assay (ELISA) kit for Horse LNPEP. The capture antibody for the commercial ELISA was a mouse monoclonal and the immunogen was a recombinant full-length horse LNPEP protein expressed by *E coli*. The detection antibody was a rabbit polyclonal antibody and the immunogen was a synthetic peptide within Human Leucyl-cystinyl aminopeptidase aa 1–50 (N terminal). National Center for Biotechnology Information (NCBI) accession: NP_005566.2. Sequence: MEPFTNDRLQLPRNMIENSMFEEEPDVVDLAKEPCLHPLEPDEVEYEPRG. The assay was performed according to the manufacturer’s instructions (MyBioSource, San Diego, CA, USA) and validated for use in our laboratory using serial dilutions of pooled serum with added amounts of the LNPEP high standard, with an intra-assay and inter-assay CV < 15%. The range of sensitivity of the assay was reported to be 6.25–200 U/L. The samples were run in duplicate using an ELISA plate reader with optical density (OD) set at 495 nm.

### ELISA for tissue LNPEP

All tissue samples from Experiment 2 and 3 were thawed, rinsed in ice-cold 0.02 mmol/L PBS pH 7.2, and minced. Tissue samples (100 mg) were manually homogenized in PBS then sonicated. The tissue was then centrifuged at 1500×*g* for 15 min. Protease inhibitor cocktail was used to avoid protein degradation (Halt™, Thermoscientific, Rockford, IL) according to user’s guide. The resulting supernatant was stored at − 80 °C and assayed at a later time. Tissue extracts were assayed for protein concentration with a commercial protein assay kit (Bio-Rad Protein Assay, BIO-RAD, Mississauga, ON) according to the manufacturer’s instructions. The same commercial ELISA (LNPEP for horses, MyBioSource, San Diego, CA) that was used for the equine serum, was used to determine the LNPEP concentration per gram (U/gr) of protein in the tissue after calculating the protein concentration.

### Western blot (WB) for OXT and LNPEP

*Sample preparation*: equine tissues were homogenized using a pre-chilled mortar and pestle method with the addition of RIPA lysis buffer supplemented with a mixture of protease inhibitors (Santa Cruz, Dallas, TX, USA) at a ratio of 1 g of tissue to 3 mL of lysis buffer. The homogenized tissues were transferred to eppendorf tubes and incubated on ice for 30 min and vortexed every 10 min. The crude total protein supernatants were obtained after centrifugation at 15,000 rpm for 10 min and were aliquoted and stored at − 20 °C until use. Equine tissues analyzed in the WB for LNPEP include: kidney, liver, ovarian follicle wall (ovary), CL, myometrium, endometrium, embryo and placenta. Equine tissued including pituitary, endometrium, myometrium and corpus luteum were used for the OXT WB.

*Determination of Protein Concentration*: the total protein concentrations from crude extracts were determined with Pierce Detergent Compatible Bradford Assay Kit (Thermo Scientific, Delaware, MA, USA) based on a standard curve obtained from a series of dilutions of bovine serum albumin (BSA) by following the kit’s instructions.

*Gel Electrophoresis and protein transfer*: the crude protein extracts (20 ug) were denatured in sample loading buffer and separated on 4–20% Mini-PROTEAN^®^ TGXTM Precast Gels with Mini-PROTEAN^®^ Tetra Cell using Tris/Glycine/SDS Electrophoresis Buffer (Bio-Rad, Hercules, CA, USA). The separated proteins were transferred onto Immobilon-P polyvinylidene difluoride (PVDF) membrane (Millipore, Bedford, MA, USA) in Tris/glycine buffer with Mini Trans-Blot® Cell (Bio-Rad, Hercules, CA, USA).

*Blocking and Antibodies*: the PVDF membranes containing transferred proteins were washed in phosphate buffered saline with 0.05% Tween 20 and incubated in blocking buffer (10% goat serum, 2xcasein in PBS) for 1 h at room temperature to prevent nonspecific binding. Primary antibodies, rabbit anti-oxytocin (ImmunoStar, Hudson, WI, USA, 1:1000), rabbit anti-LNPEP (LifeSpan BioSciences, Seattle, WA, USA, 1:1000) were diluted in PBS containing 1 × casein and incubated overnight at 4 °C. To determine epitope-specific binding, a separate PVDF membrane was performed in parallel with isotype negative control diluted to equivalent final concentration (rabbit IgG at 1:5000 for LNPEP, rabbit sera at 1:1000 for OXT). After that, the membranes were washed in PBS containing 0.05% tween 20 for 5 min for 3 times, and incubated with biotinylated goat anti-rabbit IgG (Vector Laboratories, Burlingame, CA, USA, 1:1,000 in PBS) for 30 min and followed by incubation with streptavidin conjugated to alkaline phosphatase (Vector Laboratories, Burlingame, CA, USA, 1:500 in PBS) for 20 min at room temperature. The positive staining was visualized by incubation with ImmPACT^®^ Vector^®^ Red chromogen (Vector Laboratories, Burlingame, CA, USA). Precision Plus ProteinTM Dual Color Standards (Bio-Rad, Hercules, CA, USA) were used for monitoring gel electrophoresis and confirmation of proper protein transfer. A separate blot against mouse anti-GAPDH (Santa Cruz, Santa Cruz, Dallas, TX, USA, 1:1000) was used as a loading control.

### Liquid chromatography tandem mass spectrometry (LC-MS/MS)

A Coomassie blue stained polyacrylamide gel was run side by side with the polyacrylamide gel used for WB to identify immune-positive bands identified by the antibodies used. The corresponding bands in the polyacrylamide gel of the expected molecular weight (MW) for OXT (MW of 10–17 kDa) and LNPEP (MW of 115–150 kDa) were excised and submitted for liquid chromatography tandem mass spectrometry (LC-MS/MS) for protein identification to the Proteomics Facility at the Institute of Biotechnology at Cornell University.

### In-gel trypsin digestion of SDS gel bands

The protein bands (~ 1 mm cubes) from an SDS-PAGE gel were subjected to in-gel digestion followed by extraction of the tryptic peptide as reported previously^49^. The excised gel pieces were washed consecutively with 200–400 μL distilled/deionized water followed by 50 mM ammonium bicarbonate, 50% acetonitrile and finally 100% acetonitrile. The dehydrated gel pieces were reduced with 50–400 μL of 10 mM dithiothreitol (DTT) in 100 mM ammonium bicarbonate for 1 h at 56 °C, and alkylated with 50–400 μL of 55 mM iodoacetamide in 100 mM ammonium bicarbonate at room temperature in the dark for 45 min. Following repeated wash steps as described above, the gel slices were then dried and rehydrated with trypsin (Promega), at an estimated 1:3 w/w ratio in 50 mM ammonium bicarbonate, 10% ACN, and incubated at 37 °C for 18 h. The digested peptides were extracted twice with 200 μl of 50% acetonitrile, 5% formic acid and once with 200 μl of 75% acetonitrile, 5% formic acid. Extractions from each sample were combined and filtered with 0.22 μm spin filter (Costar Spin-X from Corning) and dried to dryness in the speed vacuum. Each sample was reconstituted in 2% acetonitrile, 0.5% formic acid prior to LC-MS/MS analysis.

### Protein identification by nano LC-MS/MS analysis

The in-gel tryptic digests were reconstituted in 20 μL of 0.5% FA for nanoLC-ESI-MS/MS analysis, which was carried out using an Orbitrap FusionTM TribridTM (Thermo-Fisher Scientific, San Jose, CA) mass spectrometer equipped with a nanospray Flex Ion Source, and coupled with a Dionex UltiMate3000RSLCnano system (Thermo, Sunnyvale, CA)^50,51^. The gel extracted peptide samples (5 μL) were injected onto a PepMap C-18 RP nano trapping column (5 µm, 100 µm i.d × 20 mm) at 15 µL/min flow rate for rapid sample loading and then separated on a PepMap C-18 RP nano column (2 µm, 75 µm × 25 cm) at 35 °C. The tryptic peptides were eluted in a 60 min gradient of 5–38% acetonitrile (ACN) in 0.1% formic acid at 300 nL/min., followed by a 7 min ramping to 90% ACN-0.1% FA and an 8 min hold at 90% ACN-0.1% FA. The column was re-equilibrated with 0.1% FA for 25 min prior to the next run. The Orbitrap Fusion was operated in positive ion mode with spray voltage set at 1.6 kV and source temperature at 275 °C. External calibration for FT, IT and quadrupole mass analyzers was performed. In data-dependent acquisition (DDA) analysis, the instrument was operated using FT mass analyzer in MS scan to select precursor ions followed by 3 s “Top Speed” data-dependent CID ion trap MS/MS scans at 1.6 m/z quadrupole isolation for precursor peptides with multiple charged ions above a threshold ion count of 10,000 and normalized collision energy of 30%. MS survey scans at a resolving power of 120,000 (fwhm at m/z 200), for the mass range of m/z 375–1575. Dynamic exclusion parameters were set at 40 s of exclusion duration with ± 10 ppm exclusion mass width. All data were acquired under Xcalibur 3.0 operation software (Thermo-Fisher Scientific).

### LC-MS/MS data analysis

The DDA raw files for CID MS/MS were subjected to database searches using Proteome Discoverer (PD) 2.3 software (Thermo Fisher Scientific, Bremen, Germany) with the Sequest HT algorithm, and processing workflow for precursor-based quantification. The PD 2.3 processing workflow containing an additional node of Minora Feature Detector for precursor ion-based quantification was used for protein identification. The database search was conducted against Equus caballus NCBI Jun 2018 database also having 245,782 entries. Two-missed trypsin cleavage sites were allowed. The peptide precursor tolerance was set to 10 ppm and fragment ion tolerance was set to 0.6 Da. Variable modification of methionine oxidation, deamidation of asparagines/glutamine and fixed modification of cysteine carbamidomethylation, were set for the database search. Only high confidence peptides defined by Sequest HT with a 1% FDR by Percolator were considered for the peptide identification. The final protein IDs contained protein groups that were filtered with at least 2 peptides per protein.

The precursor abundance intensity for each peptide identified by MS/MS in each sample was automatically determined and their unique peptides for each protein in each sample were summed and used for calculating the protein abundance by PD 2.3 software without normalization.

### Immunohistochemistry (IHC) for OXT and LNPEP

The immunohistochemistry of the equine tissues (pituitary, endometrium, myometrium, corpus luteum and placenta) was performed by the Immunopathology Research and Development Core Laboratory at Cornell University. Briefly, 4-μm–thick sections of formalin-fixed/paraffin-embedded sections were used for immunohistochemical analysis. After deparaffinization in xylene and rehydration in graded ethanol, the sections were subjected to antigen retrieval by steaming in citrate buffer (10 mM, pH 6.0) for 20 min and followed by 30 min cooling at room temperature. Then, endogenous peroxidase activity was quenched by 0.3% hydrogen peroxide in distilled water for 10 min. An IHC analysis was performed by using ImmPRESS^®^ HRP Goat Anti-Rabbit IgG (Peroxidase) Polymer Detection Kit (Vector Laboratories) following the kit instruction. The tissue sections were incubated with primary antibodies for 1.5 h at room temp on an orbital shaker. For LNPEP, the rabbit polyclonal anti-human LNPEP IgG was purchased from LifeSpan BioSciences, Inc (LSBio, Cat# LS-B12918/65,145) and used at 1:200 for all tissues. For oxytocin, rabbit anti-synthetic oxytocin whole serum was purchased from ImmunoStar (cat#20,068) and used at 1:15,000 for pituitary and at 1:500 for the rest of tissues. Negative controls were run in parallel by replacing the primary antibody with rabbit IgG for LNPEP and rabbit serum for oxytocin at the equivalent final concentration. Nova Red (Vector Laboratories) was used as chromogen to visualize antigen localization, and the sections were lightly counterstained with hematoxylin. All stained IHC slides were digitized with Aperio CS2 (Leica Biosystems).

### RNA extraction and real-time polymerase chain reaction (qPCR)

Placental tissue was obtained from a subset of 7 mares from the body (B), pregnant horn (PH), and non-pregnant horn (NPH) and were utilized for qPCR analysis. Total RNA was extracted from placental samples using TRIzol Reagent (ThermoFisher Scientific, Waltham, MA) according to the manufacturer’s instructions, and RNA concentration and purity were determined using a NanoDrop spectrophotometer (ThermoFisher). Samples with 260/280 and 260/230 ratios of approximately 2 were considered pure and used for the analyses. 500 ng of RNA was treated with RNase-free DNase I and reverse transcribed into cDNA using the High-Capacity cDNA Reverse Transcription Kit (ThermoFisher) according to the manufacturer’s instructions and stored at − 20 °C until real time PCR.

The mRNA relative abundance was measured by real-time PCR for Oxytocin/neurophysin 1 (*OXT)* and leucyl and cystinyl aminopeptidase (*LNPEP*), and the sequence for the primers were used from the previous literature^52^. Quantitative PCR was run using the QuantStudio 3 real-time PCR system and PowerTrack SYBR Green Master Mix (Applied Biosystems). Total reaction volume was 10 uL, which contained 1 uL of cDNA (25 ng), 0.2 uL (200 nM) each of forward and reverse primers, 5 uL of SYBR Green master mix, and 3.6 uL of nuclease-free water. All samples were performed in duplicate along with a no reverse-transcriptase control and a no template control. The real-time PCR run included an initial denaturation and enzyme activation step (10 min at 95 °C) followed by 40 cycles of denaturation (15 s at 95 °C) and annealing (1 min at 60 °C). A melt curve was collected from 60 to 95 °C to check for non-specific amplification in each well. An electrophoresis gel was performed to confirm the correct size and purity of the amplified product. Glyceraldehyde 3-phosphate dehydrogenase (GAPDH) was used as the reference gene. Expression levels for our target genes were normalized to the expression of GAPDH to obtain the Delta Ct values.

### RNA in situ hybridization (RNAscope)

Fresh tissues (pituitary, endometrium, myometrium, and corpus luteum) were immediately fixed in 10% formalin in neutral buffered saline for 24 h at room temperature and submitted to the Histology Lab at the Animal Health Diagnostic Center at Cornell University for dehydration and embedding. Five μm–thick sections of formalin-fixed/paraffin-embedded sections were used for RNAscope ISH staining by following the instructions for “Formalin-fixed paraffin-embedded sample preparation and pretreatment” (document no. 322452, ACDBio, Newark, CA, USA). Prior to staining, all slides are baked in a drying oven for 1 h at 60 °C. After deparaffinization in xylene and rehydration in graded ethanol, the sections were subjected to antigen retrieval. For equine pituitary gland, the slides were steamed in ACD’s RNAscope Target Retrieval solution (1×) for 15 min, and then the slides were further incubated in RNAscope Protease Plus for 15 min at 40 °C. For equine corpus luteum, endometrium, and myometrium, the slides were steamed in ACD’s RNAscope Target Retrieval solution (1×) for 8 min, and then the slides were further incubated in RNAscope Protease Plus for 15 min at 40 °C. The endogenous peroxidase activity was quenched by 0.3% hydrogen peroxide in distilled water for 10 min at room temperature.

For LNPEP ISH, RNAscope 2.0 HD detection kit (Red) was used by following ACD’s user manual (Cat. No. 320487). The RNAscope LNPEP probe was designed based on 20ZZ named Ec-LNPEP targeting 2202–3146 of XM_005599529.2. RNAscope negative probe DapB was performed in parallel for assessing non-specific background staining during initial optimization process.

For oxytocin ISH, the BaseScope™ Reagent Kit v2-RED was used by following the user manual (Cat. No.323910). The BaseScope oxytocin probe was designed based on 3ZZ probe targeting sequence provided, 2–150 named BA-Ec-oxt-3zz-st. BaseScope negative probe DapB-3ZZ was performed in parallel for assessing non-specific background staining during initial optimization process.

### Statistical analysis

All data analysis was performed with JMP Pro 13.1(SAS Institute Inc.) with a *p*-value of *P* ≤ 0.05. Residual plots and Shapiro–Wilk tests were utilized to determine normality, and data was ranked transformed when necessary. For Experiment 1, serum P4 was analyzed with a mixed model (REML) with progesterone as a dependent variable with the model statement including group, day and group*day interaction. Serum LNPEP was also analyzed in the same fashion, however serum P4 was added as an effect in the model and mare was added as a random factor. For experiment 2 and 3, serum and tissue LNPEP were analyzed with Kruskal–Wallis tests with post-hoc Dunn’s test. The mRNA data was analyzed with One-Way ANOVA test.

## Supplementary Information

Supplementary Information.

## Data Availability

The mass spectrometry proteomics data have been deposited to the ProteomeXchange Consortium via the PRIDE^53^ partner repository with the dataset identifier PXD036368.

## References

[1] Chatterjee O (2016). An overview of the oxytocin-oxytocin receptor signaling network. J. Cell Commun. Signal..

[2] Gimpl G, Fahrenholz F (2001). The oxytocin receptor system: structure, function, and regulation. Physiol. Rev..

[3] Sharp DC, Thatcher MJ, Salute ME, Fuchs AR (1997). Relationship between endometrial oxytocin receptors and oxytocin-induced prostaglandin F2 alpha release during the oestrous cycle and early pregnancy in pony mares. J. Reprod. Fertil..

[4] Behrendt-Adam CY, Adams MH, Simpson KS, McDowell KJ (1999). Oxytocin-neurophysin I mRNA abundance in equine uterine endometrium. Domest. Anim. Endocrinol..

[5] Bae S-E, Watson ED (2003). A light microscopic and ultrastructural study on the presence and location of oxytocin in the equine endometrium. Theriogenology.

[6] Watson ED, Buckingham J, Björkstén TS (1999). Immunolocalisation of oxytocin in the equine ovary. Equine Vet. J..

[7] Stout TA, Lamming GE, Allen WR (1999). Oxytocin administration prolongs luteal function in cyclic mares. J. Reprod. Fertil..

[8] Vanderwall DK, Rasmussen DM, Woods GL (2007). Effect of repeated administration of oxytocin during diestrus on duration of function of corpora lutea in mares. J. Am. Vet. Med. Assoc..

[9] Keith L (2013). Diestrus administration of oxytocin prolongs luteal maintenance and reduces plasma PGFM concentrations and endometrial COX-2 expression in mares. Theriogenology.

[10] Nomura S (2005). Gene regulation and physiological function of placental leucine aminopeptidase/oxytocinase during pregnancy. Biochim. Biophys. Acta Proteins Proteomics.

[11] Nomura, S., Tsujimoto, M. & Mizutani, S. Chapter 84—Cystinyl Aminopeptidase/Oxytocinase. In: *Handbook of Proteolytic Enzymes (Third Edition)*, Third Edit., Eds: N.D. Rawlings and G. Salvesen, Academic Press. pp 419–425 (2013).

[12] Keller SR, Scott HM, Mastick CC, Aebersold R, Lienhard GE (1995). Cloning and characterization of a novel insulin-regulated membrane aminopeptidase from Glut4 vesicles. J. Biol. Chem..

[13] Tsujimoto M, Hattori A (2005). The oxytocinase subfamily of M1 aminopeptidases. Biochim. Biophys. Acta Proteins Proteomics.

[14] Mastick CC, Aebersold R, Lienhard GE (1994). Characterization of a major protein in GLUT4 vesicles. Concentration in the vesicles and insulin-stimulated translocation to the plasma membrane. J. Biol. Chem..

[15] Nakamura H (2000). Oxytocin stimulates the translocation of oxytocinase of human vascular endothelial cells via activation of oxytocin receptors. Endocrinology.

[16] Kobayashi H (2004). Tissue distribution of placental leucine aminopeptidase/oxytocinase during mouse pregnancy. J. Histochem. Cytochem..

[17] Laustsen PG (1997). The complete amino acid sequence of human placental oxytocinase. Biochim. Biophys. Acta.

[18] Yamahara N (2000). Placental leucine aminopeptidase/oxytocinase in maternal serum and placenta during normal pregnancy. Life Sci..

[19] Nagasaka T (1997). Immunohistochemical localization of placental leucine aminopeptidase/oxytocinase in normal human placental, fetal and adult tissues. Reprod. Fertil. Dev..

[20] Ito N (2003). Ultrastructural localization of aminopeptidase A/angiotensinase and placental leucine aminopeptidase/oxytocinase in chorionic villi of human placenta. Early Hum. Dev..

[21] Mizutani S, Yoshino M, Oya M (1976). A comparison of oxytocinase and L-methionine-insensitive leucine aminopeptidase during normal pregnancy. Clin. Biochem..

[22] Mizutani S (1982). Human placental leucine aminopeptidase (P-LAP) as a hypotensive agent. Experientia.

[23] Mizutani S (1985). In vitro degradation of oxytocin by pregnancy serum, placental subcellular fractions and purified placental aminopeptidases. Exp. Clin. Endocrinol..

[24] Iwase A, Nomura S, Mizutani S (2001). Characterization of a secretase activity for placental leucine aminopeptidase. Arch. Biochem. Biophys..

[25] Armstrong DL (2017). The core transcriptome of mammalian placentas and the divergence of expression with placental shape. Placenta.

[26] Hestand MS (2015). Annotation of the protein coding regions of the equine genome. PLoS ONE.

[27] Dini P (2018). Kinetics of the chromosome 14 microRNA cluster ortholog and its potential role during placental development in the pregnant mare. BMC Genom..

[28] Pham V (2009). Reproduction and maternal behavior in insulin-regulated aminopeptidase (IRAP) knockout mice. Peptides.

[29] Matsumoto H, Mori T (1998). Changes in cystine aminopeptidase (oxytocinase) activity in mouse serum, placenta, uterus and liver during pregnancy or after steroid hormone treatments. Zoolog. Sci..

[30] Zavy MT, Bazer FW, Sharp DC, Wilcox CJ (1979). Uterine luminal proteins in the cycling mare. Biol. Reprod..

[31] Ikenaga H, Ono K, Tomoda I (1994). Elution profiles and molecular weights of placental cystine aminopeptidase in animals. J. Vet. Med. Sci..

[32] Ikenaga H (1993). Placental and plasma cystine aminopeptidase in pregnant animals. J. Vet. Med. Sci..

[33] Mustafa T, Chai S-Y, May CN, Mendelsohn FAO, Albiston AL (2004). Oxytocinase/insulin-regulated aminopeptidase is distributed throughout the sheep, female reproductive tract and is regulated by oestrogen in the uterus. Regul. Pept..

[34] Toda S (2002). Existence of placental leucine aminopeptidase/oxytocinase/insulin-regulated membrane aminopeptidase in human endometrial epithelial cells. J. Clin. Endocrinol. Metab..

[35] Kotani Y, Iwase A, Ando H, Mizutani S (2005). Oxytocin-induced prostaglandin E2 (PGE2) synthesis is regulated by progesterone via oxytocinase in Ishikawa cells. Horm. Metab. Res..

[36] Ikenaga H (1993). Changes of plasma cystine aminopeptidase activities in pregnant mares. Jpn. J. Equine Sci..

[37] Wilkins PA (2003). Monitoring the pregnant mare in the ICU. Clin. Tech. Equine Pract..

[38] Diel de Amorim M, Bramer SA, Rajamanickam GD, Klein C, Card C (2002). Serum progesterone and oxytocinase, and endometrial and luteal gene expression in pregnant, nonpregnant, oxytocin, carbetocin and meclofenamic acid treated mares. Theriogenology.

[39] Piotrowska-Tomala KK (2022). The effects of prostaglandin E2 treatment on the secretory function of mare corpus luteum depends on the site of application: An in vivo study. Front. Vet. Sci..

[40] Vanderwall DK, Rasmussen DM, Carnahan KG, Davis TL (2012). Effect of administration of oxytocin during diestrus on corpus luteum function and endometrial oxytocin receptor concentration in cycling mares. J. Equine Vet. Sci..

[41] Diel de Amorim M, Bramer SA, Rajamanickam GD, Klein C, Card C (2002). Endometrial and luteal gene expression of putative gene regulators of the equine maternal recognition of pregnancy. Anim. Reprod. Sci..

[42] Loux SC, Dini P, El-Sheikh Ali H, Kalbfleisch T, Ball BA (2019). Characterization of the placental transcriptome through mid to late gestation in the mare. PLoS ONE.

[43] Nakanishi Y (2000). Immunoaffinity purification and characterization of native placental leucine aminopeptidase/oxytocinase from human placenta. Placenta.

[44] MacLean EL (2019). Challenges for measuring oxytocin: The blind men and the elephant?. Psychoneuroendocrinology.

[45] Rodgers RJ, O’Shea JD, Findlay JK, Flint APF, Sheldrick EL (1983). Large luteal cells the source of luteal oxytocin in the sheep. Endocrinology.

[46] Kinsey CG (2007). Constitutive and ligand-induced nuclear localization of oxytocin receptor. J. Cell. Mol. Med..

[47] McCracken, J. A., Custer, E. E. & Lamsa, J. C. Luteolysis: a neuroendocrine-mediated event. *Physiol. Rev.* **79**, 263–323 (1999). 10.1152/physrev.1999.79.2.263 10221982

[48] Diel de Amorim M (2019). Comparison of foaling prediction technologies in periparturient standardbred mares. J. Equine Vet. Sci..

[49] Yang Y, Thannhauser TW, Li L, Zhang S (2007). Development of an integrated approach for evaluation of 2-D gel image analysis: impact of multiple proteins in single spots on comparative proteomics in conventional 2-D gel/MALDI workflow. Electrophoresis.

[50] Thomas CJ, Cleland TP, Zhang S, Gundberg CM, Vashishth D (2017). Identification and characterization of glycation adducts on osteocalcin. Anal. Biochem..

[51] Yang Y, Anderson E, Zhang S (2018). Evaluation of six sample preparation procedures for qualitative and quantitative proteomics analysis of milk fat globule membrane. Electrophoresis.

[52] Diel de Amorim M (2022). Expression of oxytocin/neurophysin I and oxytocinase in the equine conceptus from day 8 to day 21 post-ovulation. Anim. Open Access J. MDPI.

[53] Perez-Riverol Y (2022). The PRIDE database resources in 2022: A hub for mass spectrometry-based proteomics evidences. Nucleic Acids Res..

