# Supplementary material for: Characterization of serum and tissue oxytocinase and tissue oxytocin in the pregnant and non-pregnant mare
Source: Sci Rep. 2023 Mar 21;13:4616. doi: 10.1038/s41598-023-31540-9 (PMC10030782; doi:10.1038/s41598-023-31540-9)
Supplement: Supplementary file 1 — Supplementary Information. [file 41598_2023_31540_MOESM1_ESM.pdf]

SDS-PAGE Coomassie blue stained gels and western blots for oxytocin-neurophysin (OXT) and oxytocinase/ leucyl and cystinyl aminopeptidase (LNPEP)

A series of images are presented from SDS-PAGE Coomassie blue stained and western blot gels for OXT and LNPEP (Pages S1 – 11)

Supplementary Figure 1

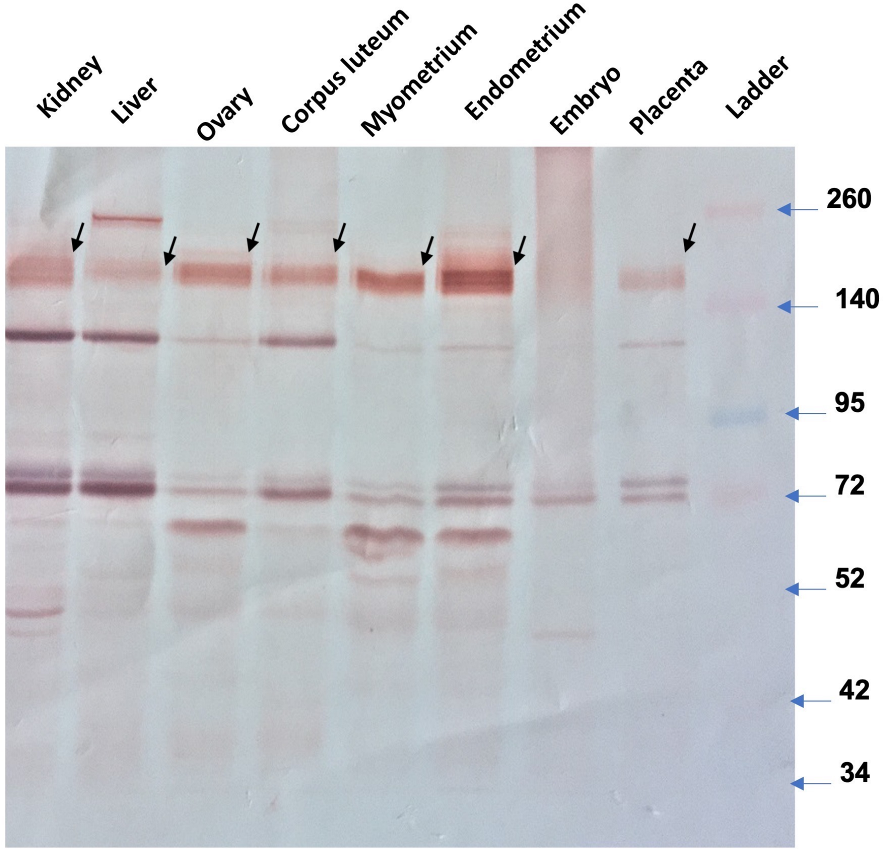

Figure 1A

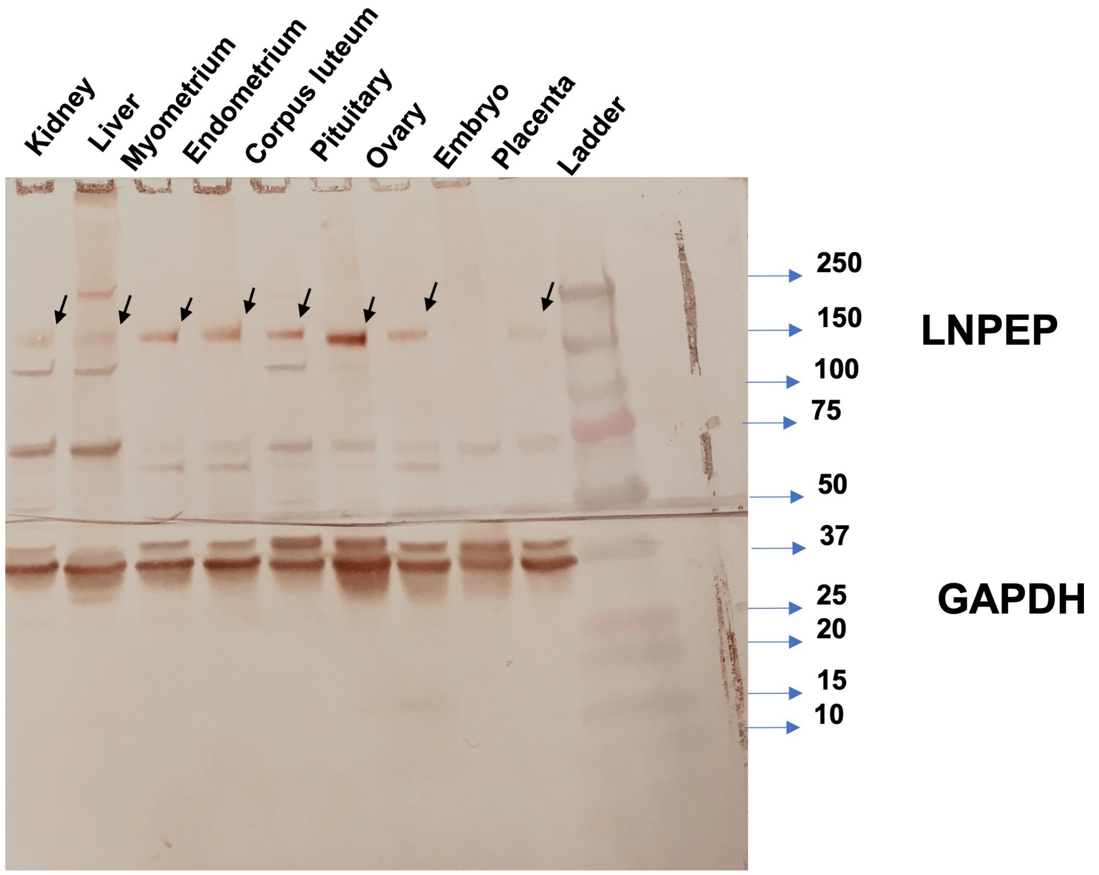

Figure 1B

Supplementary Figure 1 Page S1 – Two western blots prepared using rabbit-LNPEP at 1:1,000, with the upper portion demonstrating immunopositive bands at the expected molecular weight (~150 kDa) for the tissues investigated (Fig 1A, 1B) and on the right bottom portion Fig 1B, mouse anti-GAPDH (37 kDa) 1:1,000 as loading control (Fig 1B). Lane labels are located at the top of the gel, and ladder and molecular weights on the right-hand side of the gel, with black arrows showing the location of the bands.

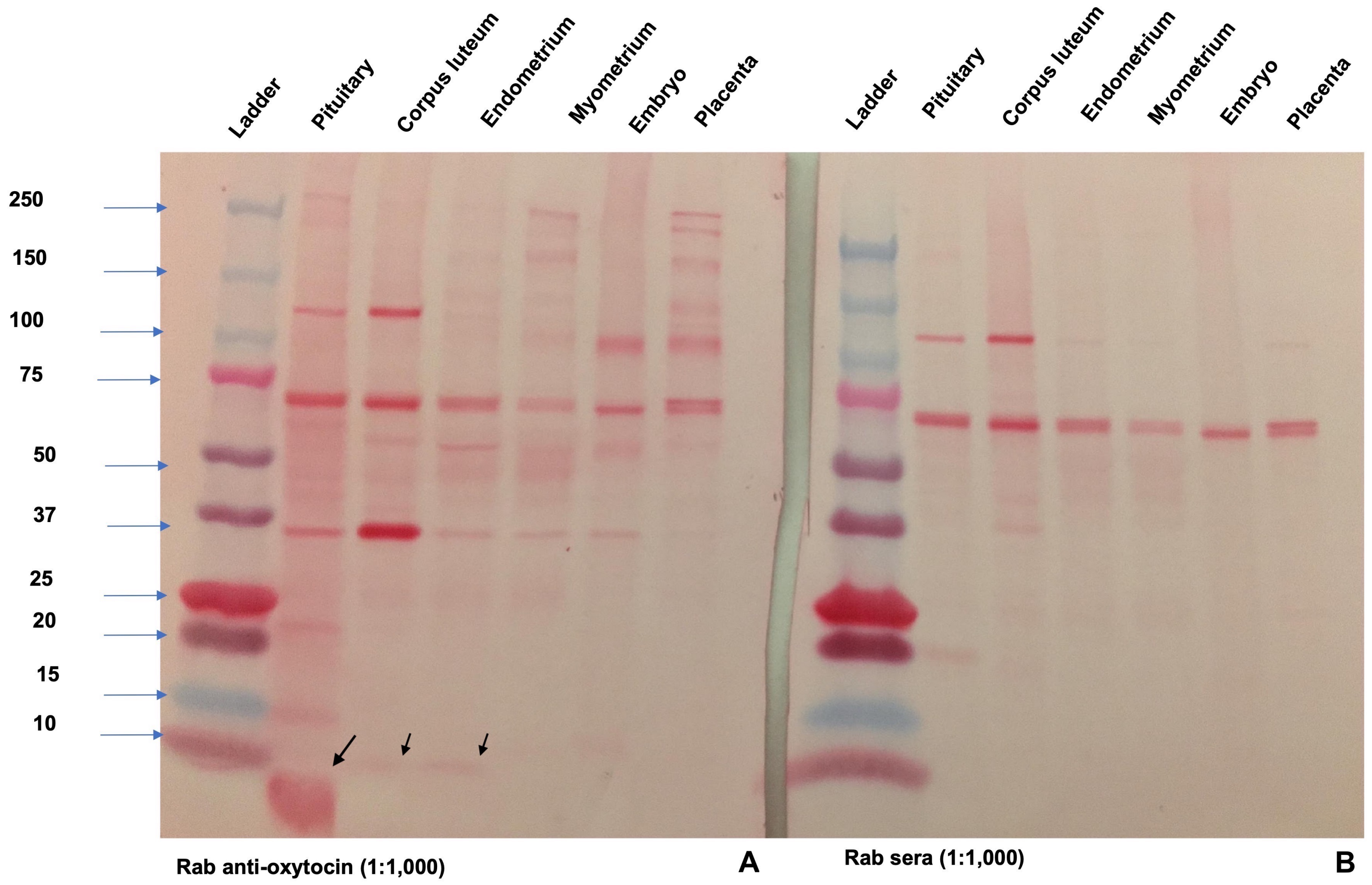

Supplementary Figure 1 Page S2 - Western blot prepared using rabbit anti-OXT at 1:1,000 demonstrating an immunopositive band at the expected molecular weight (~10 kDa) for pituitary and weak staining for the CL (A), and a separate PVDF membrane was performed in parallel with isotype negative control (B). Lane labels are located at the top of the gel, and ladder and molecular weights on the left-hand side of the gel, with black arrows showing the location of the bands. No immunostaining for OXT was noted in 1B at the expected molecular weight for the tissue extracts.

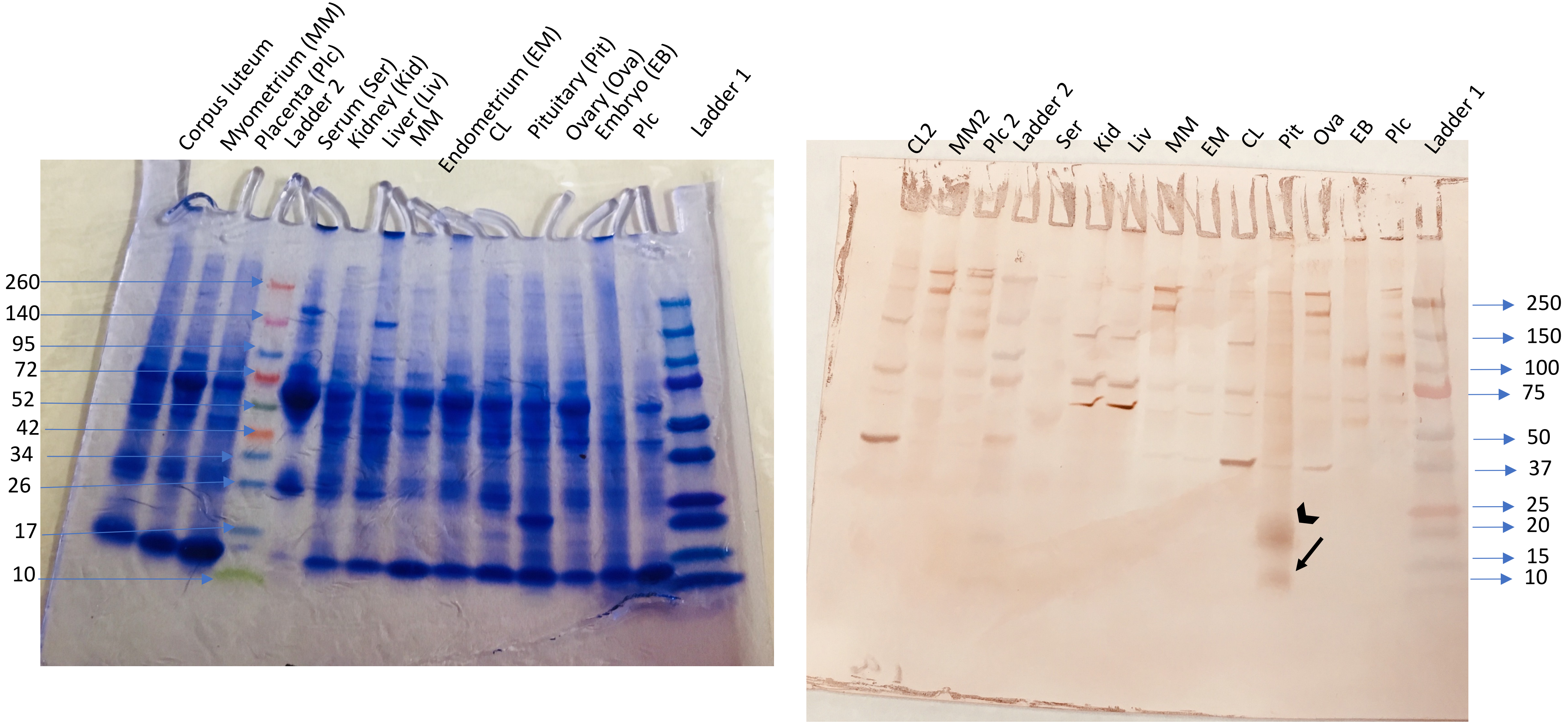

Supplementary Figure 1 Page S3 - Figure shows images of two gels; 4-20% SDS-PAGE Coomassie Blue (left), and a western blot using rabbit anti-oxytocin at 1:1,000 (right). Lane labels and abbreviations for the tissues are in parentheses at the top. Images show pituitary OXT immunostaining at a10 kDa band (black arrow), diffuse immunostaining at 17 kDa (black arrowhead). Corpus luteum (CL), myometrium (MM), Placenta (Plc), Serum (Ser), Kidney (Kid), Liver (Liv), endometrium (EM), Pituitary (Pit), Ovary (Ova), Embryo (EB).

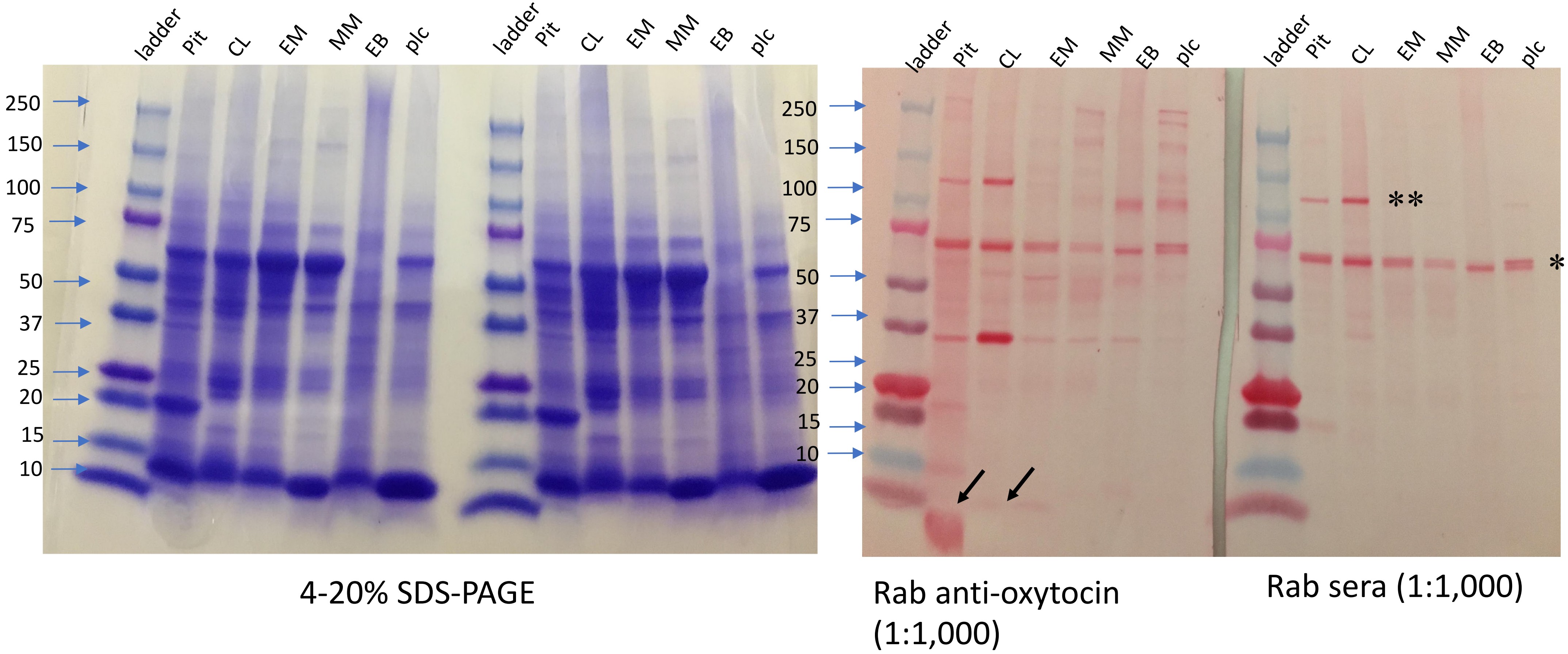

Supplementary Figure 1 Page S4 - Figure shows four gels from left to right: two 4 – 20% SDS PAGE gels stained with Coomassie blue, and two OXT western blots prepared with rabbit anti-oxytocin and negative control rabbit sera at 1:1000. Lane labels for the tissues are at the top. In gel 3 there is OXT immunostaining for bands in the pituitary and CL at 10kDa (black arrows). The 4th gel is a western blot prepared with the corresponding rabbit serum control at 1:1,000. There are non-specific immunostained bands at 75kDa (all tissues, this lane denoted by an *) and 110 kDa (pituitary and CL denoted by **). Corpus luteum (CL), myometrium (MM), Placenta (Plc), Serum (Ser), Kidney (Kid), Liver (Liv), endometrium (EM), Pituitary (Pit), Ovary (Ova), Embryo (EB).

Oxytocin gel separation – excised bands for LC-MS/MS analysis before vs after

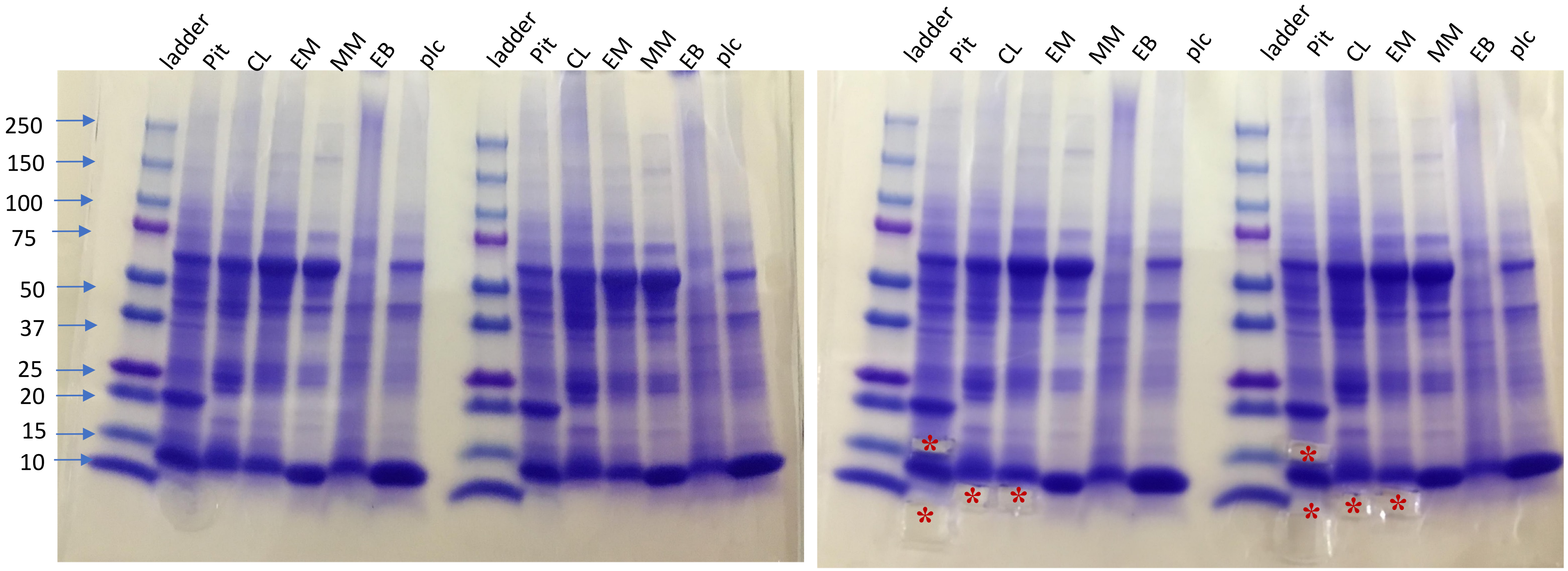

Excised: Pit10, Pit20, CL10, EM10, combined 2 lanes, 40ug/lane

Supplementary Figure 1 Page S5 - Figure shows four 4 – 20% SDS PAGE gels stained with Coomassie Blue. The gels are loaded with 40 µg of homogenized tissue extract The two gels on the left-hand side are intact (before band excision), and the two gels on the right show the following excision of the bands (denoted by *) corresponding to the molecular weight of OXT which were submitted for LC-MS/MS. Lane labels for the tissues are at the top. Corpus luteum (CL), myometrium (MM), Placenta (Plc), Serum (Ser), Kidney (Kid), Liver (Liv), endometrium (EM), Pituitary (Pit), Ovary (Ova), Embryo (EB).

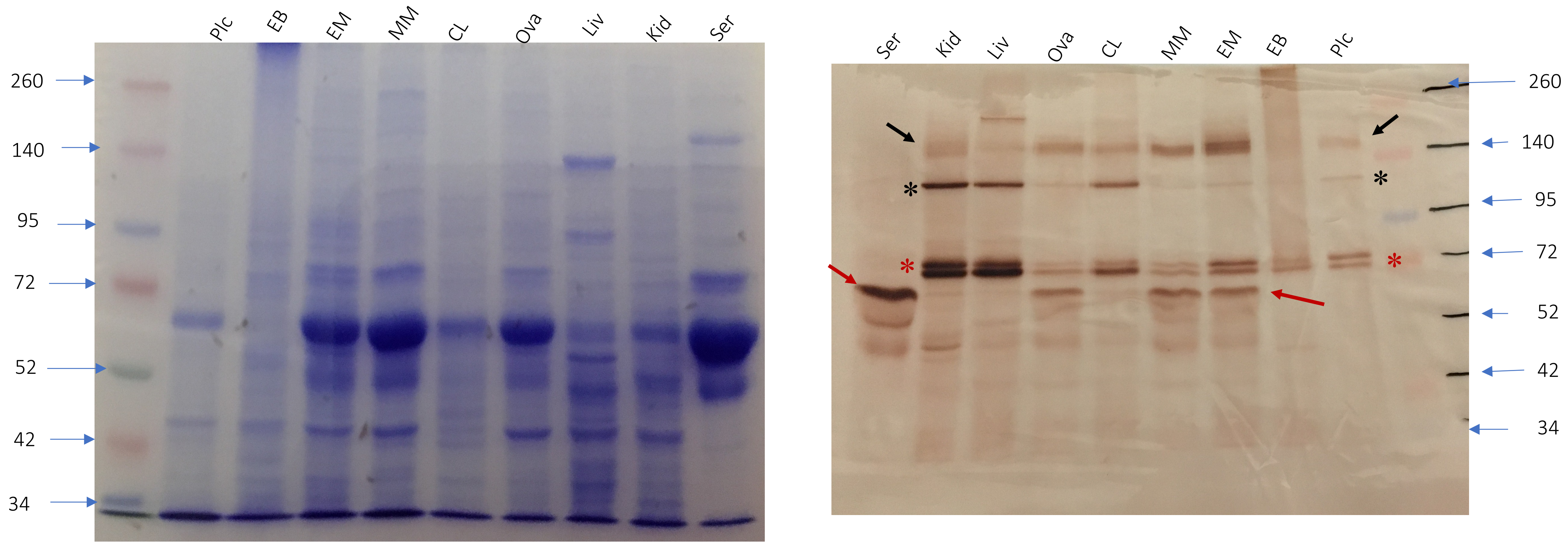

Supplementary Figure 1 Page S6 - Figure shows image of a 7.5% SDS-PAGE gel stained with Coomassie Blue (left) and western blot (right) prepared with rabbit anti-LNPEP at 1:1,000. Lane labels for the tissues are at the top. The intensity of the LNPEP immunostaining was subjectively graded from 0 (no immunostaining) to 5 (intense immunostaining). Immunostaining was identified between the black arrows at 150 kDa in kidney (kid) (2+), liver (liv) (1+), ovary (ova) (3+), corpus luteum (CL) (2+), myometrium (MM) (3+), endometrium (EM) (3+), and placenta (Plc) (1+). Immunostaining was identified between black asterisks at 120 kDa in kidney (5+), liver (5+), CL (3+), myometrium (3+), endometrium (1+), and placenta (1+). Additional immunostained bands were identified between red asterisks at 72 kDa in kidney (5+), liver (5+), ovary (1+), CL (3+), myometrium (1+), endometrium (3+), embryo (2+) and placenta (2+), and between red arrows at 65kDa serum (5+), ovary (2+), myometrium (2+) and endometrium (2+).

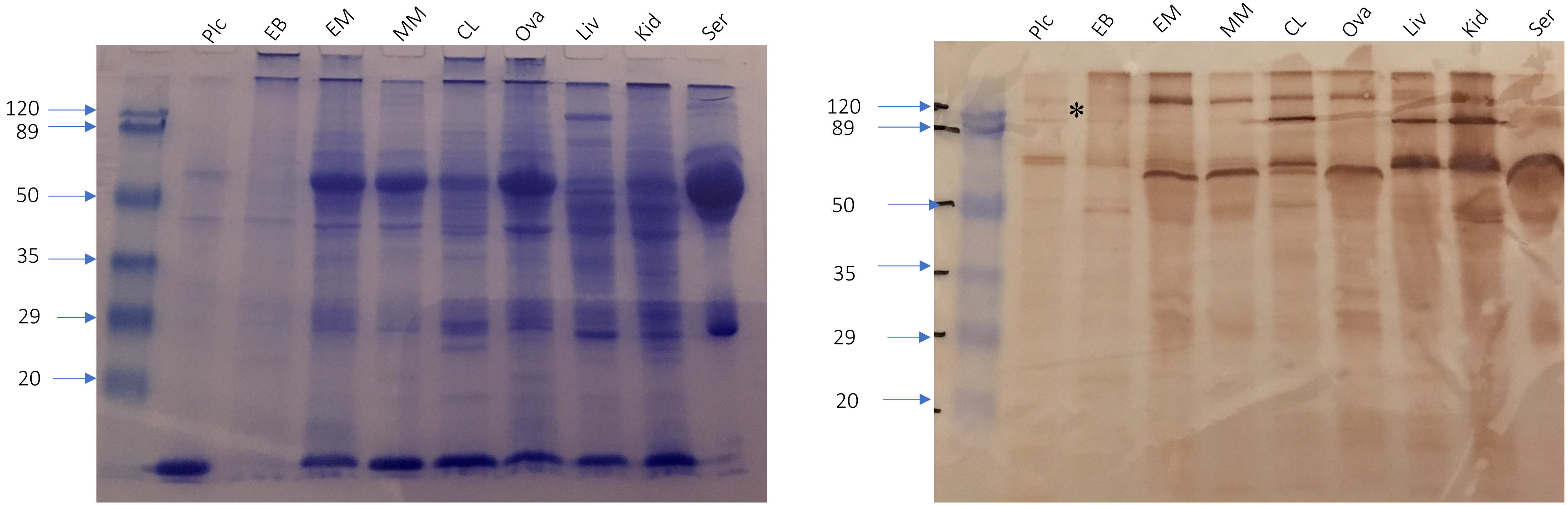

Dec 15, 2018: 12 % SDS PAGE

Supplementary Figure 1 Page S7 - Figure shows images of a 12% SDS - PAGE gel stained with Coomassie Blue (left) and western blot (right) prepared with rabbit anti-LNPEP at 1:1,000. Lane labels for the tissues are at the top and the molecular weight ladder is in lane 1 on both gels. The immunostaining in the western blot shown in previous blot (Page S1) and a faint band in placenta is present (asterisks). Corpus luteum (CL), myometrium (MM), Placenta (Plc), Serum (Ser), Kidney (Kid), Liver (Liv), endometrium (EM), Pituitary (Pit), Ovary (Ova), Embryo (EB).

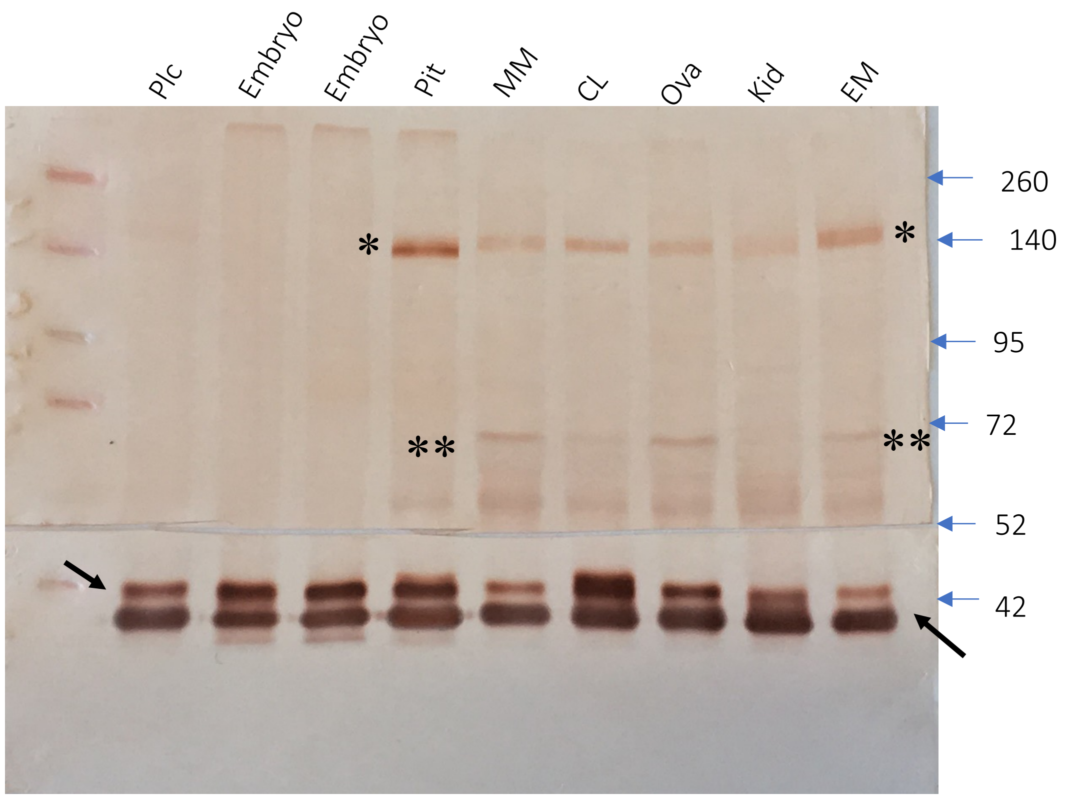

May 1, 2019: 7.5 % SDS-PAGE, Gel#2, same sample preps as 4/25/19, denatured and stored at -20°C

Positive bands: ~150 kD in Pit (4+), MM (3+), CL (3+), Ova (3+), Liv (2+), Kid (2+), EM (3+)

Positive bands: ~65 kD in Ser (4+), Ova (3+), MM (2+), EM(2+)

Supplementary Figure 1 Page S8 - The figure shows an image of a 7.5% SDS-PAGE western blot using homogenized denatured tissue extracts stored at -20°C and rabbit anti-LNPEP at 1: 1,000. The lanes are labelled at the top. The molecular weight ladder is in lane 1. There were immunopositive positive bands at 150 kDa (*) for: pituitary (4+), myometrium (3+), CL (3+), ovary (3+), liver (2+), kidney (2+), endometrium (3+) and positive bands (**) at ~65 kDa in: serum (4+), ovary (3+), myometrium (2+), endometrium (2+). A band (4+) appears at around 42 kDa in the various tissues (arrows). The immunopositive bands identified in previous gels at 120kDa and 72 kDa are faint or no longer visible and no band is evident for the placenta. Corpus luteum (CL), myometrium (MM), Placenta (Plc), Serum (Ser), Kidney (Kid), Liver (Liv), endometrium (EM), Pituitary (Pit), Ovary (Ova), Embryo (EB).

LNPEP – gel separation vs Western blot

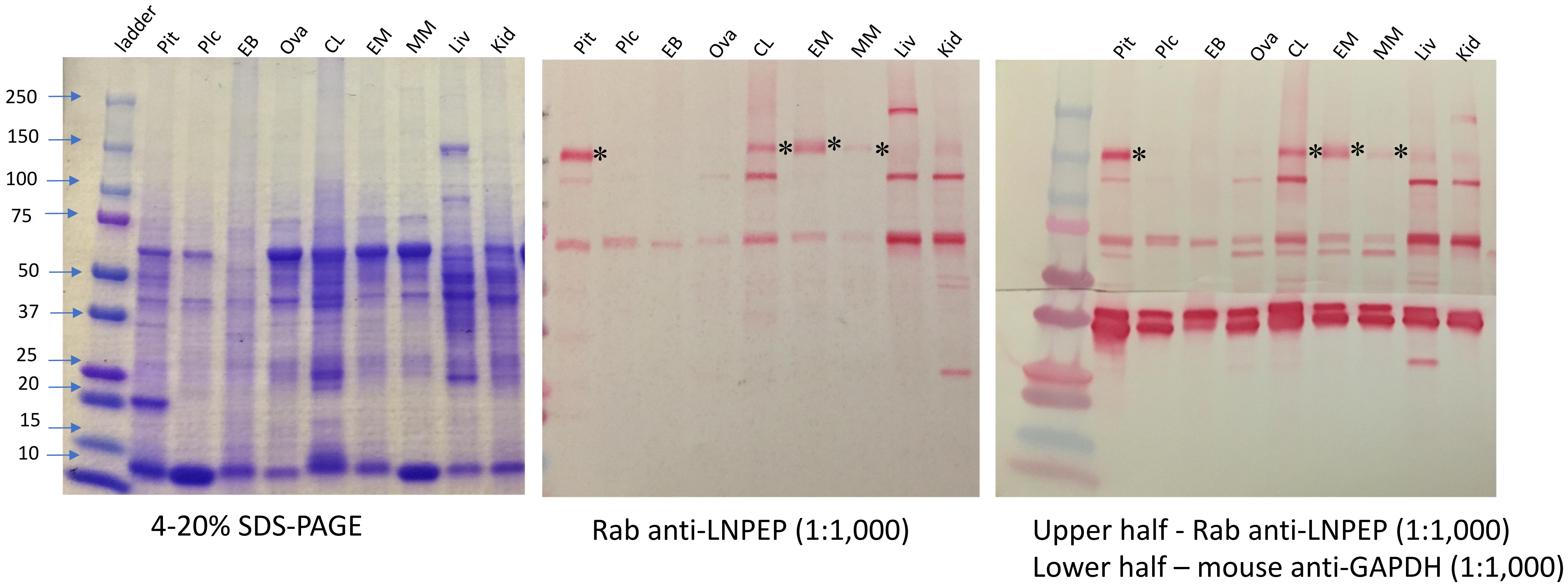

Supplementary Figure 1 Page S9 - The figure shows an image of a 4 - 20% SDS-PAGE Coomassie blue stained gel (left) and two western blots using of homogenized denatured tissue extracts stored at -20°C with rabbit anti-LNPEP at 1:1,000 in the middle gel and the gel on the right with rabbit anti-LNPEP 1:1,000 (on the upper half) and mouse anti-GADPH at 1:1,000 (on the lower half). The lanes are labelled at the top. The molecular weight ladder is in lane 1. There were LNPEP specific immunopositive positive bands at 150 kDa in: pituitary (3+), endometrium (2+), CL (2+) and myometrium (1+) (asterisks), but not in placenta. Corpus luteum (CL), myometrium (MM), Placenta (Plc), Serum (Ser), Kidney (Kid), Liver (Liv), endometrium (EM), Pituitary (Pit), Ovary (Ova), Embryo (EB).

LNPEP Western blot – antibody vs isotype negative control

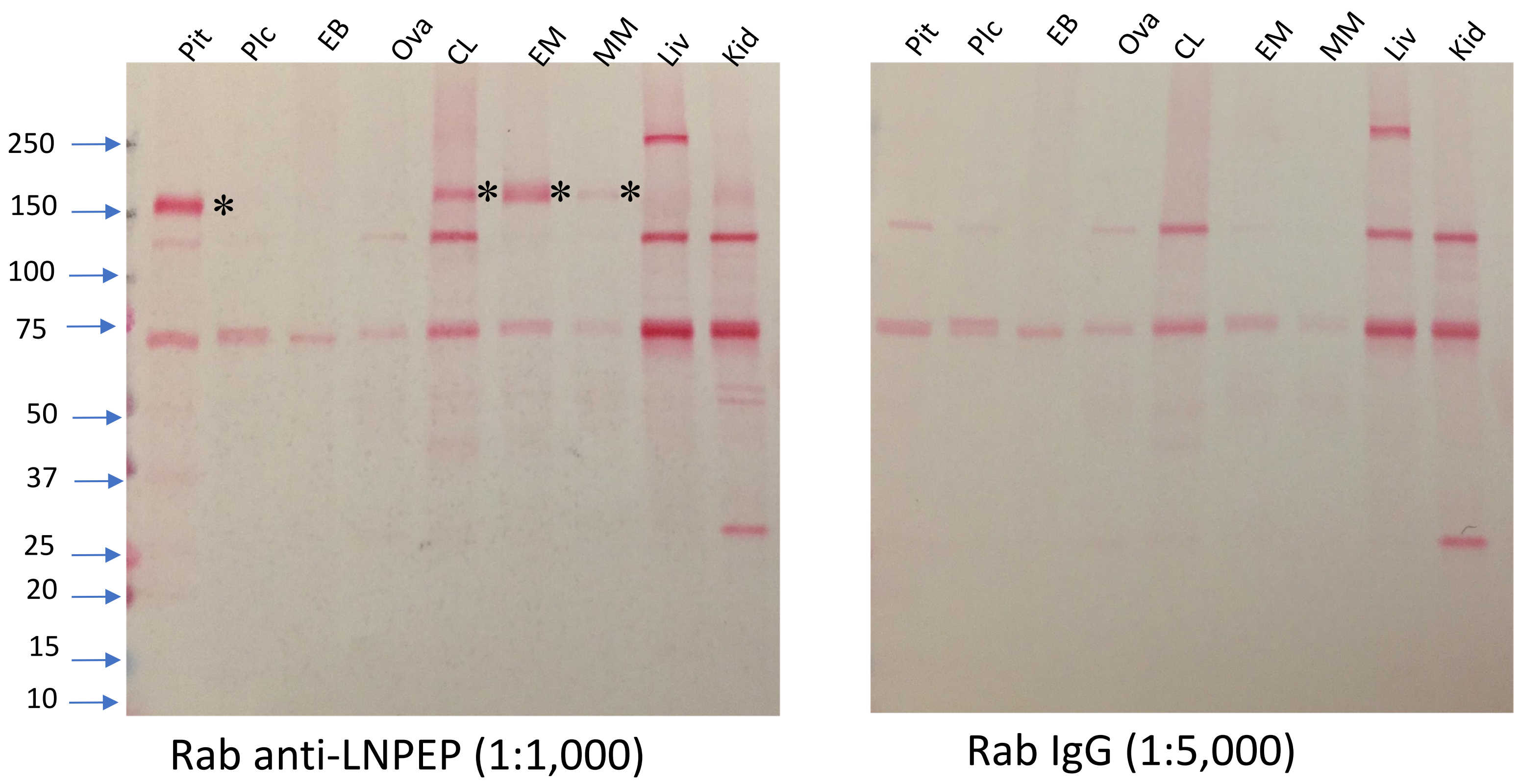

Supplementary Figure 1 Page S10 - The figure shows an image of two western blots using homogenized denatured tissue extracts stored at -20°C and rabbit anti-LNPEP at 1:1,000 (left) and rabbit IgG 1:5,000 (right) as an isotype negative control. The lanes are labelled at the top and the molecular weight marker is in lane 1. There were LNPEP specific immunopositive positive bands at 150 kDa in: pituitary (3+), endometrium (2+), CL (2+), and myometrium (1+) (asterisks), but no immunostained placenta. Corpus luteum (CL), myometrium (MM), Placenta (Plc), Serum (Ser), Kidney (Kid), Liver (Liv), endometrium (EM), Pituitary (Pit), Ovary (Ova), Embryo (EB).

LNPEP gel separation – excised bands for LC-MS/MS analysis before vs after

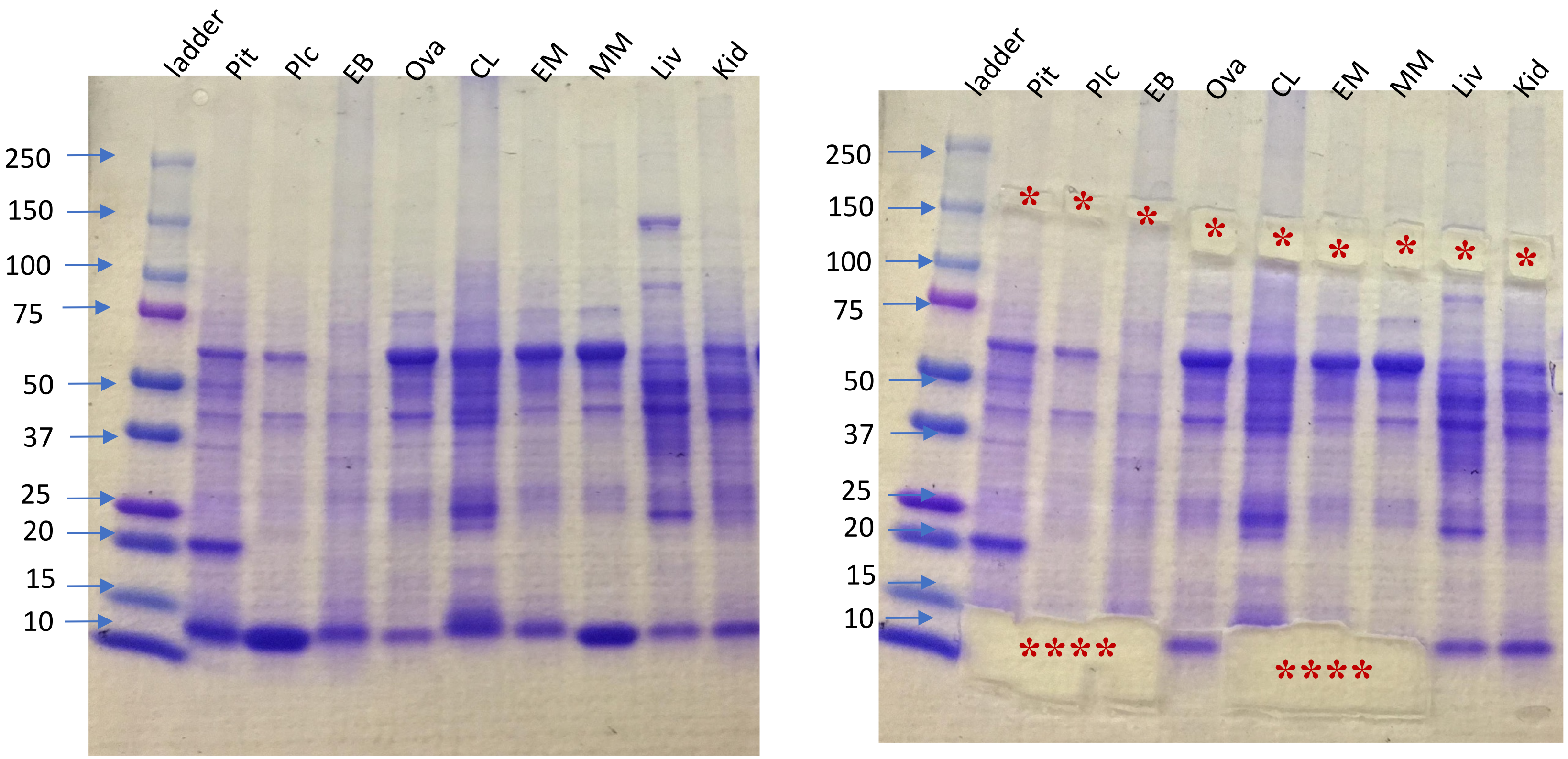

Supplementary Figure 1 Page S11 - The figure is an image of two Coomassie blue gels using 20 µg of homogenized denatured tissue extracts stored at -20°C. The lanes are labelled at the top and the molecular weight markers are in lane 1 on both gels. The figures show the gel before (left) and after (right) bands were excised (red asterisks) from the lanes for LC-MS/MS analysis. This included at 150 kDa: pituitary, placenta and embryo combined; 120 – 150 kDa pooled ovary, CL, endometrium, myometrium, liver and kidney; 10-15 kDa pituitary, placenta and embryo, and combined CL, endometrium and myometrium. Corpus luteum (CL), myometrium (MM), Placenta (Plc), Serum (Ser), Kidney (Kid), Liver (Liv), endometrium (EM), Pituitary (Pit), Ovary (Ova), Embryo (EB).
